# Time-dependent density functional theory calculations of the solvatochromism of some azo sulfonamide fluorochromes

**DOI:** 10.1007/s00894-015-2651-z

**Published:** 2015-04-16

**Authors:** Przemysław Krawczyk

**Affiliations:** Collegium Medicum, Department of Physical Chemistry, Nicolaus Copernicus University, Kurpińskiego 5, 85-950 Bydgoszcz, Poland

**Keywords:** Density functional theory, Electronic excited state, Solvatochromism, Two-photon absorption spectra, Fluorescence spectra, Fluorochrome

## Abstract

The absorption and emission spectra of three azo sulfonamide compounds in different solvents were investigated theoretically by using response functions combined with density functional theory (DFT), while the solvent effect on the structure and the electronic transitions was determined using the integral equation formalism for the polarizable continuum model (IEF-PCM). The results show that the applied different exchange-correlation functionals can reproduce the experimental values well. DFT calculations of the title compounds showed that the *H*-bond formed between the solute and solvent molecules is one of the major causes of the reversible solvatochromism observed in measured spectra. This is due to a better stabilization of the neutral form than the zwitterionic form in the polar protic solvents, which is characteristic of the hypsochromic shift. On the other hand, the molecules considered exhibit a monotonic behavior regarding the polarity of the low-lying excited state (Δμ_g–CT_) as a function of the solvent polarity. This dependence occurs in the case of the positive solvatochromism and confirms the thesis regarding the *H*-bond solute–solvent interactions. Theoretically determined values of the two-photon cross section revealed that the (*σ*_OF_^(2)^) shows similar trends with changes in *λ*_abs_, in contrast to 〈*δ*^OF^〉 values. In conclusion, the results demonstrate that the investigated molecules can be used successfully as fluorochromes in bioimaging.

**Electronic supplementary material:**

The online version of this article (doi:10.1007/s00894-015-2651-z) contains supplementary material, which is available to authorized users.

## Introduction

The pharmacological activities of sulfonamide derivates have been well known for many years [1]. The past decade has seen renewed interest in sulfonamides as potential medications. In addition to continuing research on antibacterial activity, and on hypoglycemic and diuretic sulfonamides [2–7], this interest is also related to other biological activities and is strictly dependent on structure. Most sulfonamides exhibit significant toxicity, which is the reason for their limited use and limits their application in favor of faster and safer antibiotics. The literature cites sulfonamides as selective COX-2 inhibitors, bradykinin B_2_ receptor antagonists, receptor selective antagonists of 5-HT_7_, receptor selective antagonists endothelin ET_A_, receptor ligands serotonin 5-HT_6_ or selective inhibitors of bacterial collagenase. However, most of these reports are related to studies on the synthesis of new sulfonamides and their effect on cancer or HIV. Studies on bacteriostatic and antidiabetic sulphonamides led to publications (1988–1992) regarding the synthesis and antitumor activity of *N*-diarylsulfonylureas [8, 9] and 4-amino-*N*-(5-chloroquinoxalin-2-yl) benzenesulfonamide [10, 11]. On the other hand, in 1996 studies began on 5,5,11-trioxo-10*H*-pyrrolo[1,2 -b] [1, 2, 5] benzothiadiazepine (PBTDs), 1-(benzenesulfonyl)-1*H*-pyrrole and 1-(benzenesulfonyl)-1*H*-indole [12, 13] with activity directed against HIV-1.

Beyond their biological activity, sulfonamide derivatives exhibit interesting features such as nonlinear optical (NLO) properties [14]. There is growing interest in using organic materials for NLO devices, functioning as second harmonic generators, frequency converters, electro-optical modulators, etc. because of the large second-order electric susceptibilities of organic materials. Noncentrosymmetrical organic systems demonstrating significant non-linear responses as high hyperpolarizability or two-photon absorption cross section (2PA) are those containing both electron-donating (D) and electron-accepting (A) substituents interacting via a system of π-conjugated double bonds [15–21]. In the case of sulfonamides, the electron-withdrawing group is the sulfonyl group [22, 23]. Compounds with such characteristics are particularly sought after in molecular imaging. Nowadays, bioimaging has become a useful tool in biological research because it offers an unique approach to visualization of the morphological details of cells [24]. Imaging of phenomena occurring at the molecular or intracellular level is possible by the introduction of live organism markers. The binding of such markers to tissue components allows for dimensional assessment of these structures, as well as monitoring of the processes taking place in them. In the dynamic and developing field of molecular imaging, studies are performed using optical methods, and the markers used are dyes emitting fluorescence [25]. Currently, the most studied fluorescent markers in bioimaging include fluorescent proteins, organic dyes, metal complexes, and semiconductor nanocrystals [26–29]. In order to obtain the best markers in one- and two-photon imaging, studies on the structural and optical properties of markers are required. These include the surface modification of fluorophore structure, e.g., with bright fluorescence, high photostability, large Stokes’ shift, high fluorescence quantum yield and flexible processability, all of which help to conjugate the fluorophore with biomolecules [30]. A whole variety of organic compounds meet all these criteria. However, sulfonamides, particularly when azo-functionalized, may also constitute a new group of fluorochromes in addition to their applications mentioned above. Azobenzene derivatives containing intramolecular D-π-A charge-transfer chromophores show excellent photo-physical properties since they have extensive π-systems delocalized between the acceptor and donor units across the azo linkage [31, 32]. This type of structure provides an acentrosymmetric distribution of electronic charge and relatively high polarity of the excited states. The molecule, which is then placed in an external electric or optic field, is subjected to electronic charge movement, causing a change in electron density, and the consequent change in polarization state causes the appearance of NLO properties [33, 34].

In the past decade, density functional theory (DFT) has been used frequently to describe the structural parameters of the ground and excited states, vibrational frequencies and energies of D-π-A types molecules [35–38]. Moreover, the calculated values obtained with this method are efficient and accurate with respect to the evaluation of a number of molecular properties [39, 40]. The DFT is used not only in the search for new materials used in photonics, but also in the theoretical modeling of drug design. Some of the most frequently used functionals for this purpose are B3LYB [41] and PBE0 [42]. However, despite their popularity, they are known to poorly predict excitation energies of the charge-transfer (CT) states [43]. In order to improve the quality of the predictions of traditional functionals, long-range corrected functionals have been proposed recently [44]. Among them, the most promising are the CAM-B3LYP and LC functionals.

In the case of the azo sulfonamide dyes investigated here, to the author’s knowledge no detailed geometry of the excited state or two-photon absorption cross section obtained during theoretical calculations is available to date. This paper also presents the characteristics of the excited states of the azo sulfonamides discussed. To confirm the adequacy of DFT methods, the theoretical one-photon absorption and fluorescence spectra will be compared with experimental values. The results of calculations of the two-photon absorption cross section will then be presented and the usefulness of these compounds as fluorescence markers will be evaluated. All cited experimental values of these molecules were determined by Zakerhamidi et al. [45].

## Calculation methods

The molecular geometries of molecules csidered here in their ground and excited states were optimized using the Gaussian 09 program package [46] and the hybrid exchange-correlation B3LYP [41], O3LYP [47], PBE0 [42, 48], PBEh1PBE [49] as well as long-range corrected CAM-B3LYP [43], LC-BLYB [44, 50], LC-ωPBE [50–52] and ωB97XD [53] functionals in conjunction with the 6–311++G(d,p) basis set. Geometry optimization was carried out both in the gas phase and in the presence of selected solvents by applying the integral equation formalism for the polarizable continuum model (IEF–PCM) [54]. In all cases, harmonic vibrational analysis was performed in order to confirm that the stationary points correspond to minima on the potential energy surface.

Using time-dependent density functional theory (TD-DFT) [36–38] with all the above-mentioned functionals, spectroscopic parameters characterizing one-photon excitation spectra were determined. In the case of excited states, the geometric parameters and fluorescence spectra were determined based on the PBE0, CAM-B3LYP and LC-ωPBE functionals. These calculations employed the Gaussian 09 suite of programs and the 6-311++G(d,p) basis set, carried out both in vacuo and with the inclusion of solvent effects using the IEF-PCM model. In this study, an approximation was made that vertical excitation energy is comparable to experimental band maxima. The former quantity was computed neglecting the effects of molecular vibrations.

In order to determine the excited state dipole moments, the dipole moment differences between ground (*g*) and excited (*e*) state were calculated by numerical differentiation of the excitation energies:

1 $$ \varDelta {\mu}_{ge}^i=\frac{E_e\left(+{F}^i\right)-{E}_e\left(-{F}^i\right)}{-2{F}^i}-\frac{E_g\left(+{F}^i\right)-{E}_g\left(-{F}^i\right)}{-2{F}^i} $$

where the index *i* denotes the Cartesian component of the dipole moment difference. An electric field *F* of strength 0.001 a.u. was used in all calculations. The numerical stability of *Δμ* calculations was checked by comparing the analytical dipole moments from ground state calculations with the values obtained by numerical differentiation of the ground state energy.

Experimentally, the two-photon absorption (2PA) can be obtained by dissipation of the incident light, which, for a single beam 2PA experiment, is twice the transition rate. In this case, the two-photon cross-section of the degenerate process is written as [55–57]:

2 $$ {\upsigma}_{OF}^{(2)}=\frac{8{\pi}^3{\alpha}^2{\hslash}^3}{e^4}\cdot \frac{\omega^2g\left(\omega \right)}{\varGamma_F/2}\left\langle {\delta}^{OF}\right\rangle $$

where *α* is a fine structure constant, *ω* is the frequency of absorbed photons (assuming one source of photons), *Γ*_F_ is the broadening of the final state (F) due to its finite lifetime and g(*ω*) provides the spectral line profile—often assumed to be a *δ*-function—and 〈*δ*^OF^〉 is the two-photon transition probability for the transition from the ground state to a final state.

In the case of a molecule absorbing two photons of the same energy in isotropic media, the degenerate 〈*δ*^OF^〉 in an isotropic medium using a linearly polarized laser beam given by [58]:

3 $$ {\delta}^{OF}=\frac{1}{15}{\displaystyle \sum_{ij}}\left[{S}_{ii}^{OF}{\left({S}_{jj}^{OF}\right)}^{*}+2{S}_{ij}^{OF}{\left({S}_{ij}^{OF}\right)}^{*}\right] $$

In this equation,* S*_ij_^OF^ is the second-order transition moment given by:

4 $$ {S}_{ij}^{OF}\left({\zeta}_1,{\zeta}_2\right)=\frac{1}{\hslash }{\displaystyle \sum_K\left[\frac{\left\langle 0\left|{\zeta}_1\cdot {\overset{\frown }{\mu}}_i\right|K\right\rangle \left\langle K\left|{\zeta}_2\cdot {\overset{\frown }{\mu}}_j\right|F\right\rangle }{\omega_{\alpha }-{\omega}_1}+\frac{\left\langle 0\left|{\zeta}_2\cdot {\overset{\frown }{\mu}}_i\right|K\right\rangle \left\langle K\left|{\zeta}_1\cdot {\overset{\frown }{\mu}}_j\right|F\right\rangle }{\omega_{\alpha }-{\omega}_2}\right]} $$

where *ℏω*_1_ + *ℏω*_2_ should satisfy the resonance condition and $\left\langle 0\left|{\zeta}_1\cdot {\overset{\frown }{\mu}}_i\right|K\right\rangle$ stands for the transition moment between electronic states |0 > and |K>, respectively. *ζ* is the vector defining polarization of photons.

To describe the two-photon allowed states, the quadratic response functions formalism [59, 60] within the DFT framework was used as implemented in the DALTON 2011 program [61, 62]. Solvent effects were taken into account with the self-consistent reaction field (SCRF) model. All 2PA calculations were carried out employing the CAM-B3LYP functional and the 6-311++G(d,p) basis set.

## Results and discussion

### Geometries of the ground state and the second excited state

**Fig. 1a–c:**
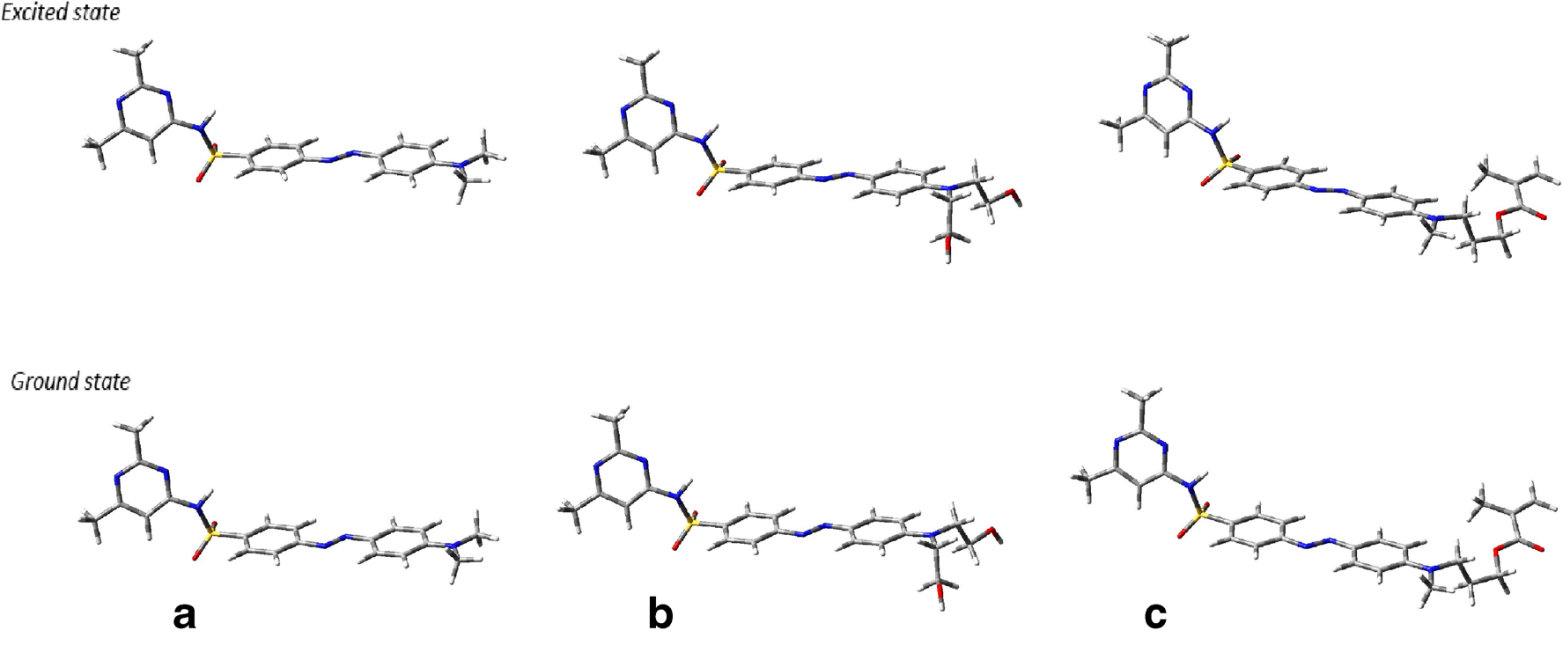
Chemical structure of the investigated molecules in their ground and lowest-lying singlet excited states obtained after optimization in G09.** a** BS1 ((*E*)-4-((4-(dimethylamino) phenyl]diazenyl)-*N*-(2,6-dimethylpyrimidin-4-yl)benzenesulfonamide).** b** BS2 ((*E*)-4-((4-(bis(2-hydroxyethyl)amino)phenyl)diazenyl)-*N*-(2,6-dimethylpyrimidin-4-yl)benzenesulfonamide).** c** BS3 ((E)-3-((4-((4-(*N*-(2,6-dimethylpyrimidin-4-yl)sulfamoyl]phenyl)diazenyl)phenyl) (methyl) amino)propyl-methacrylate)

**Fig. 2:**
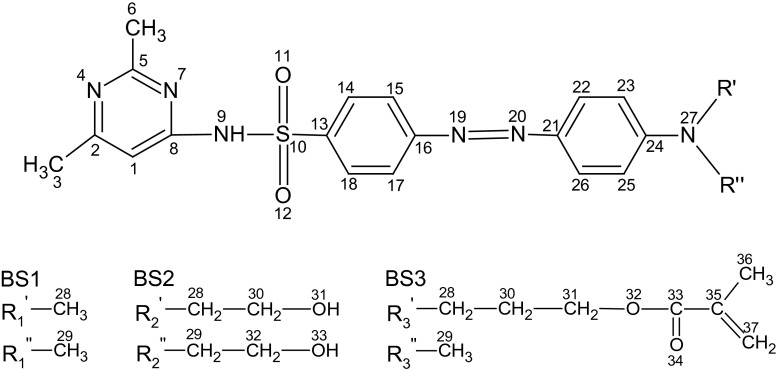
A method of numbering the atoms forming the tested azo sulfonamide dyes

For the investigated azo sulfonamide derivatives (cf. Figs. 1, 2), the excitation of the lowest energy is of n–π* character (dark state) while intense charge-transfer excitation is observed for the second excited singlet state (bright state), accompanied by the largest oscillator strength. The selected optimized parameters of the ground (*S*_0_) and the second excited (*S*_2_) state are listed in Tables 1 and 2. The complete characterization of geometrical parameters can be seen in the Supporting Information. In addition, the PBE0 functional was used to optimize the structures and the plots of frontier orbitals also with the 6-311++G(d,p) basis set. This functional was chosen as the absorption spectra were closest to experimental values, as discussed below.

**Table 1:** Selected bond lengths determined by time-dependent density functional theory (TD-DFT) at PBE0/6-311++G(d,p) level of theory with inclusion of solvent effects. Values in Ångstroms.* DMSO* Dimethlysulfoxide

	BS1				BS2				BS3			
	Gas phase	Acetone	DMSO	Water	Gas phase	Acetone	DMSO	Water	Gas phase	Acetone	DMSO	Water
8–9	1.407/1.398	1.406/1.393	1.406/1.395	1.406/1.396	1.408/1.388	1.407/1.395	1.406/1.392	1.406/1.393	1.408/1.403	1.406/1.388	1.406/1.394	1.406/1.395
9–10	1.715/1.699	1.708/1.695	1.707/1.697	1.707/1.698	1.714/1.693	1.708/1.695	1.707/1.703	1.707/1.704	1.715/1.721	1.709/1.735	1.708/1.696	1.708/1.700
10–11	1.461/1.459	1.466/1.466	1.466/1.465	1.466/1.464	1.461/1.454	1.466/1.473	1.466/1.491	1.466/1.469	1.461/1.462	1.466/1.470	1.466/1.467	1.467/1.467
10–12	1.460/1.458	1.465/1.464	1.465/1.464	1.465/1.464	1.460/1.455	1.465/1.463	1.465/1.463	1.465/1.463	1.460/1.462	1.465/1.469	1.465/1.468	1.465/1.468
10–13	1.789/1.754	1.782/1.745	1.782/1.740	1.782/1.733	1.790/1.755	1.783/1.742	1.783/1.737	1.783/1.742	1.789/1.773	1.782/1.710	1.782/1.768	1.782/1.743
16–19	1.416/1.344	1.412/1.341	1.412/1.339	1.412/1.345	1.416/1.365	1.414/1.342	1.414/1.341	1.415/1.343	1.416/1.375	1.416/1.403	1.415/1.402	1.412/1.404
19–20	1.261/1.247	1.267/1.247	1.268/1.248	1.268/1.249	1.260/1.303	1.265/1.244	1.266/1.246	1.266/1.248	1.261/1.296	1.267/1.205	1.268/1.217	1.268/1.218
20–21	1.396/1.351	1.387/1.351	1.387/1.354	1.386/1.377	1.397/1.402	1.390/1.354	1.389/1.356	1.389/1.357	1.396/1.422	1.387/1.333	1.386/1.357	1.386/1.362
24–27	1.372/1.373	1.361/1.360	1.361/1.362	1.360/1.361	1.382/1.382	1.373/1.375	1.373/1.375	1.373/1.375	1.375/1.387	1.364/1.356	1.363/1.348	1.363/1.349
27–28	1.456/1.443	1.461/1.444	1.461/1.447	1.461/1.448	1.461/1.447	1.464/1.450	1.464/1.449	1.464/1.449	1.460/1.457	1.464/1.454	1.464/1.453	1.464/1.448
27–29	1.457/1.444	1.462/1.343	1.462/1.449	1.462/1.447	1.461/1.446	1.464/1.450	1.465/1.443	1.465/1.440	1.457/1.454	1.463/1.452	1.463/1.451	1.463/1.447
30–31	–	–	–	–	1.425/1.406	1.430/1.411	1.430/1.404	1.430/1.402	1.448/1.435	1.452/1.437	1.453/1.436	1.453/1.438
32–33	–	–	–	–	1.425/1.406	1.430/1.411	1.430/1.404	1.430/1.401	1.357/1.350	1.345/1.342	1.345/1.342	1.344/1.342

**Table 2:** Selected bond and torsion angles determined by TD-DFT at PBE0/6-311++G(d,p) level of theory with inclusion of solvent effects. Values expressed as degrees

	BS1				BS2				BS3			
	Gas phase	Acetone	DMSO	Water	Gas phase	Acetone	DMSO	Water	Gas phase	Acetone	DMSO	Water
8–9–10	124.139/124.517	124.016/124.385	124.119/124.414	124.166/124.424	124.209 128.085	124.082/124.549	124.125/124.993	124.137/124.761	124.053/125.581	124.037/124.167	124.048/125.336	124.051/124.127
9–10–13	99.550/99.986	100.230/101.078	100.284/100.843	100.302/100.749	99.559/106.306	100.231/100.509	100.261/101.107	100.281/101.618	99.620/100.235	100.298/103.023	99.957/101.852	100.369/101.048
9–10–11	110.314/110.500	110.597/109.652	110.615/109.766	110.620/109.987	110.375/102.876	110.620/110.170	110.650/109.704	110.656/109.929	110.216/109.402	110.552/108.274	110.561/106.651	110.564/106.593
9–10–12	106.400/106.671	106.504/105.306	106.513/105.369	106.515/105.394	106.419/106.446	106.535/105.876	106.543/104.878	106.545/104.237	106.435/105.886	106.459/103.399	106.469/103.385	106.472/103.345
15–16–19	124.712/124.451	124.893/121.935	124.903/122.299	124.907/123.940	124.699/124.435	124.835/121.811	124.830/121.983	124.832/122.441	124.704/124.358	124.859/121.672	124.87/121.689	125.089/121.774
17–16–19	115.493/118.395	115.530/118.626	115.530/118.359	115.531/117.200	115.471/116.548	115.477/118.557	115.494/118.369	115.494/118.032	115.496/116.514	115.549/116.677	115.651/116.695	115.703/116.745
16–19–20	114.591/130.833	114.613/132.505	114.620/132.187	114.622/130.907	114.626/114.531	114.677/130.650	114.658/130.643	114.656/130.638	114.615/115.405	114.602/125.972	114.609/125.138	114.611/124.528
19–20–21	116.284/130.18	116.690/131.129	116.95/131.207	116.99/131.246	116.210/119.573	116.719/129.901	116.774/129.647	116.784/128.305	116.250/118.211	116.864/133.932	116.895/130.533	116.904/130.394
20–21–22	116.311/118.645	116.324/118.297	116.326/118.254	116.326/117.247	116.418/117.059	116.422/118.857	116.399/120.860	116.399/119.409	116.381/116.622	116.395/118.697	116.399/117.916	116.400/117.026
20–21–26	125.296/124.080	125.510/122.701	125.521/123.216	125.525/124.543	125.377/124.840	125.536/122.768	125.568/122.710	125.572/122.416	125.343/124.968	125.551/121.996	125.560/122.337	125.562/122.420
1–8–9–10	35.635/33.549	34.004/33.505	33.940/33.251	33.916/33.018	35.378/30.271	34.367/30.811	33.952/30.749	33.915/30.657	36.411/33.502	33.335/34.301	33.267/35.632	33.242/35.352
7–8–9–10	−146.924/-148.781	−148.465/-149.438	−148.531/-149.399	−148.555/−149.288	147.160/−151.763	148.072/−151.381	148.421/−151.235	148.458/−151.191	−146.214/−148.993	−148.977/−147.385	−149.037/−147.409	−149.040/−147.921
8–9–10–11	76.424/73.277	71.868/70.717	71.771/70.645	71.755/70.583	76.793/75.899	70.478/69.542	70.179/69.351	70.175/69.841	76.560/80.676	69.112/65.160	68.838/65.504	68.760/65.917
8–9–10–12	−55.286/−54.24	−57.867/−56.802	−58.872/−56.996	−59.857/−57.526	54.966/45.742	−59.297/−57.612	−59.501/−57.867	−59.471/−59.196	55.219/50.177	60.527/61.536	60.697/61.320	60.742/61.240
8–9–10–13	−169.857/−173.030	−174.906/−179.666	−173.204/−175.138	−173.197/−174.200	169.508/169.894	174.517/176.649	174.754/176.323	174.783/175.944	−169.726/−172.194	−175.841/−178.245	−176.043/−176.185	−176.099/−176.109
16–19–20–21	179.946/177.855	179.176/176.494	179.901/176.620	179.901/176.636	179.712/−178.729	179.809/−175.460	179.868/−172.624	179.877/−170.615	−179.821/−179.482	179.977/−177.194	179.986/−177.91	179.993/−172.036

As seen in Table 1, most bond lengths in the ground state are longer than those in the excited state. It is easy to see that the major changes occur in bonds connecting the aromatic rings, while the* S*_2_ state bond lengths of pyrimidine and azobenzene rings are practically identical to those in the* S*_0_ state. For each of the investigated molecules, the binding of N19–N20 is reduced much more than 0.015 Å. However, in the case of BS1 and BS2 compounds, the shortening of the C16–N19 bond is greater, while the NN double bond approaches more towards the ring connected to the sulfonic group. However, for BS3 molecule, the nitrogen–nitrogen π-bridge is closer to the third ring, connected to the amino group. This results in a significant approach of the pyrimidine ring to the azobenzene moiety. The geometrical parameters are also influenced by the presence of different amino groups, which are characteristic for each of the discussed molecules. Taking into account the N27–C28 and N27–C29 bond lengths, which are reduced, it can be assumed that, in the case of tertiary amines, the first alkyl carbon atoms will be closing in the direction of a nitrogen atom, while practically no change will occur for the bond linking the aromatic fragment with the N atom. An interesting observation is also provided by analysis of the behavior of oxygen bonds linking it with other elements. As can be seen, the double bonds show no changes while single bonds are shortened by more than 0.012 Å. These structural changes occur in two different groups, hydroxy and ether for BS2 and BS3, respectively. Thus, in the case of other groups in which an oxygen atom will be attached via single or double bonds; an analogous dependence is to be expected when excitation occurs.

The presence of a solvent effect significantly influences the geometry of the ground and excited state. In the case of the aminosulfane group, which is linked to the fragment of the pyrimidine ring with azobenzene, C8–N9, N9–S10 and S10–13 bonds in the ground state are shortened during the transition from the gas phase into acetone. However, a further transition to more polar solvents is not accompanied by a change in length of these bonds. In turn, when comparing the effect of solvent on the differences between the lengths of these bonds in the* S*_0_ and* S*_2_ state (Δ*S*_0_–*S*_2_), it is easy to see that, in the case of BS1, the difference in the gas phase is the smallest and does not exceed 0.016 Å. During the transition to acetone, Δ*S*_0_–*S*_2_ increases up to 0.072 Å for the S10–C13 bond. The presence of a more polar solvent results in a progressive reduction of these differences; however, for water they are still higher than in vacuum. In the case of BS2 and BS3, for the C8–N9 and N9–C10 bonds in the gas phase, Δ*S*_0_–*S*_2_ is larger than in the presence of solvents, and increasing the dielectric constant of the medium promotes larger differences. However, in the case of S10–C13, Δ*S*_0_–*S*_2_ is significantly greater in solution than in vacuo, and upon excitation it is shortened greatly. This causes the sulfur atom to shift further toward the azobenzene fragment, rather than toward the pyrimidine ring. Particularly sensitive to environmental changes is the NN π-bridge. In any case, not only is the NN bond reduced during the excitation, but the value of Δ*S*_0_–*S*_2_ is also decreased with increasing polarity of the medium. Furthermore, in the case of BS1 and BS2, the presence of more polar solvents tends to increase the value of ΔS_0_–S_2_ for C16–N19 bond and reduce it for N20–C21, which causes the NN double bond to approach the ring with amino group. However, for the BS3 molecule, an inverse correlation is observed. This suggests that increasing the length of alkyl groups in the aromatic amine linked to the π-electron bridge causes it to approach a part of the sulfonamide.

The structural changes that take place when the studied azo sulfonamides are excited to the second excited state are caused not only by the change in bond lengths between the atoms. The planar location of the different fragments forming the molecules is also significant. As seen from Table 2, the values of the C8–N9–S10 and N9–S10–C13 torsion angles of the ground and excited states rise as a function of the polarity of the medium. On the other hand, during the excitation of molecules there is observed a reduction in these angles for each case. Moreover, a trend highlights that, with increasing polarity of the medium, the size of these angles decreases. These observations indicate that in the S_2_ state, the overall length of the sulfamyl connector is shorter; thereby, the pyrimidine is closer to the azobenzene fragment. These changes occur also in the dihedral angle. In the case of BS1 and BS2 molecules, the C1–C8–N9–S10 angle decreases, and the C7–C8–N9–S10 increases. This is caused by twisting of the pyrimidine ring around the C8–N9 bond, whereby it is arranged more perpendicularly to the symmetry axis of the azobenzene molecules than in the case of the ground state. This rotation is in the direction of the oxygen atom O12. Therefore, in the excited state, the reduction of C8–N9–O12–S10 angle is greater than the C8–N9–O12–S10. This observation is analogous in all analyzed media wherein, with an increase in dielectric constant, these structural changes are reduced. For the BS3 molecule, the opposite relation is observed: the position of the pyrimidine ring moves more in the direction of the axis of symmetry of azobenzene. Changes in the geometrical parameters are also visible in the azo bridge. As seen from Table 2, the values of angles C15–C16–N19 and N20–C21–C26 for* S*_0__and_* S*_2_ rise gradually with increasing dielectric constant of the medium. However, for all molecules, the Δ*S*_0_–*S*_2_ values for C15–C16–N19 and N20–C21–C26 are more than 2°. Simultaneously, the angles C16–C17–N19 and N20–C21–C22 are reduced by more than 1°. In addition, an increase of more than 10° is observed for C16–N19–N20 and N19–N20–C21. In contrast, the dihedral angle C16–N19–N20-N20–C2 turns out to be smaller for* S*_0_ relative to* S*_2_. On the other hand, these changes cause a slight stretch of the length of the *π*-electron bridge during the excitation. In the solution, increasing the polarity of the medium results in a decrease of the Δ*S*_0_–*S*_2_ values. Some discrepancies are observed for the BS3. This may be induced by the presence of an expanded amino group which, crucially, may influence the size of the charge transfer, but also the structural parameters.

**Fig. 3:**
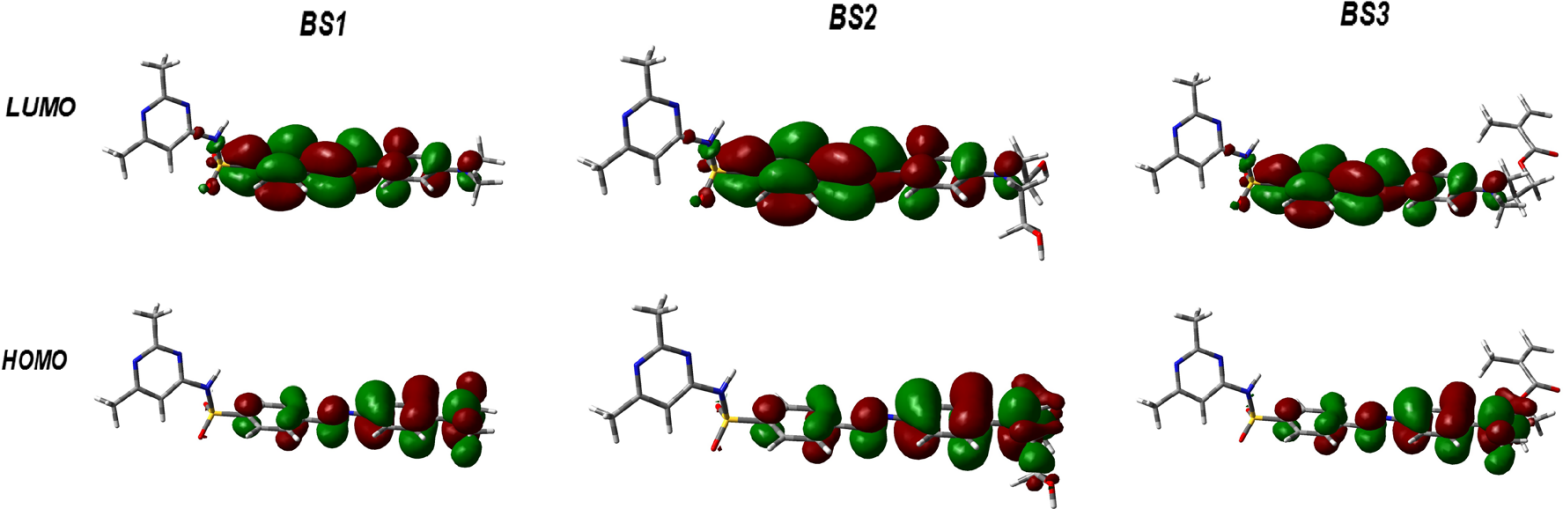
Plots of orbital contour surfaces for investigated compounds. The molecular orbitals were calculated at the PBE0/6-311++G(d,p) level of theory in vacuum. Contour surfaces of orbital amplitude 0.02 (*red*) and −0.02 (*green*) are shown

As mentioned earlier, for the titled azo sulfonamides, an intense charge-transfer excitation is observed for the second excited singlet state (π–π*) and this transition is dominated by highest occupied molecular orbital to lowest unoccupied molecular orbital (HOMO→LUMO) excitation (see Fig. 3). The figures show that both HOMO and LUMO electrons are delocalized on the sulfamyl linker, on azobenzene and on the nitrogen atom of the amine group. In each case, the pyrimidine ring is omitted. The HOMO is localized mainly on the part of the molecule that corresponds to the π-electron bridge and one of azobenzene rings with the attached nitrogen atom of the amino group. The lowest-lying unoccupied molecular orbital is localized on the second ring of azobenzene and the sulfamyl linker. Therefore, during the excitation to the* S*_2_ state, there is a substantial shift in electron density distribution from the amino group in the direction of the sulfamyl linker and this constitutes an intramolecular charge transfer, reducing the D-π-A form. On the other hand, the presence of the pyrimidine ring with electron-donating groups (methyl groups) does not affect the size of this transfer. Both frontier molecular orbitals are localized mainly on the azobenzene rings, indicating that the HOMO–LUMO transitions involve mostly π-antibonding type orbitals. The value of the energy separation between these orbitals is 3.084, 2.919 and 3.077 eV for BS1, BS2 and BS3, respectively. These relatively large values of the HOMO-LUMO gap suggest high excitation energies for many of the excited states, a good stability and a high chemical hardness for the investigated azo sulfonamides.

### Electronic absorption and emission spectra

The electronic absorption spectra (*λ*_abs_), oscillator strengths (f_OS_) and experimental UV–vis spectra calculated in gas phase and solvents with different DFT functionals for BS1, BS2 and BS3 are shown in Tables 3, 4 and 5, respectively. Comparison of the eight functionals used in evaluation of one-photon absorption (OPA) bands indicates that the long-range corrected (LC) functionals give similar results. The values obtained using LC functionals are practically identical, with the differences between them being smaller than 4 nm. Slightly higher values were obtained using LC-*ω*PBE. In contrast, the use of CAM-B3LYP leads to the maxima of the absorption bands shifting towards longer wavelengths, and the difference Δ*λ*_CAM-B3LYP - LC-BLYP_ exceeds 35 nm. Lastly, *ω*B97XD, which includes empirical dispersion, predicts the discussed quantity at the CAM-B3LYP level and the difference between them is less than 7 nm, with the latter giving a slightly higher value. On the other hand, the use of another group, which comprises standard hybrid functionals, leads to much higher *λ*_abs_. The relatively often used B3LYP overestimates these values by more than 50 and 80 nm with respect to CAM-B3LYP and LC-BLYP, respectively. In turn, both PBE functionals lead to *λ*_abs_ values at the same level (Δ*λ*_PBEh1PBE - PBE0_ ≈ 0.3 nm), and the values obtained using these functionals are 10 nm lower relative to B3LYP, but 30 nm higher compared to CAM-B3LYP. More importantly, comparison of calculated and experimental values indicates that the most reliable values were obtained with PBE functionals. This method gives values of transition energy (ΔE) that are slightly overestimated relative to experimental values but the average difference of ΔΔE between them is only 0.11 eV for BS1, 0.06 eV for BS2 and 0.12 eV for BS3. The asymptotically corrected CAM-B3LYP and LC functionals give underestimated results compared with those obtained experimentally. Here, the average deviation is 0.15 and 0.24 eV for CAM-B3LYP, 0.41 and 0.52 eV for LC, in the case of the BS1 (and BS3) and BS2, respectively. In the case of *ω*B97XD functional, as long as it provides absorption maximum bands at a similar level to those of CAM-B3LYP, the ΔΔE values obtained with its use are significantly larger, i.e., 0.20 eV for BS1 and BS3, and 0.29 eV for BS2. This is in agreement with the results obtained for azobenzene derivatives and diarylethane photochromes presented by Jacquemin [63]. It has been shown that, for singlet states, the *ω*B97XD results are similar to their CAM-B3LYP counterparts, and that this range-separated functional including dispersion is very efficient for this family of compounds. Similar relationships for ketocyanine dyes were observed by Eilmes [64] and for benzoic acid derivatives by Guo et al. [65]. Moreover, the Yang group [66] showed that this functional provided relatively accurate descriptions for charge-transfer excitation for platinum arylacetylide complexes. In the case of the latter two hybrid functionals there is a significant overestimation, with the differences being 0.17, 0.11 and 0.18 eV for B3LYP, 0.25, 0.20 and 0.26 eV for O3LYP, in the case of BS1, BS2 and BS3. Despite these significant differences, the use of these two functionals leads to more reliable results, as they give values less affected by errors than in the case of LC functionals. This indicates clearly that, for this type of molecule, new testing methods are still required.

**Table 3:** Calculated and experimental values of excitation energies (*λ*_abs_ in nm) and oscillator strengths (f_os_) for the BS1 molecule.* DMF* Dimethlyformamide

BS1	B3LYP		CAM-B3LYP		LC-BLYP		LC-*ω*PBE		O3LYP		PBE0		PBEh1PBE		*ω*B97XD		Exp.
	*λ*_abs_	f_os_	*λ*_abs_	f_os_	*λ*_abs_	f_os_	*λ*_abs_	f_os_	*λ*_abs_	f_os_	*λ*_abs_	f_os_	*λ*_abs_	f_os_	*λ*_abs_	f_os_	*λ*_abs_
Gas phase	421.69	1.15	377.12	1.28	345.59	1.31	348.38	1.32	437.64	1.09	409.93	1.20	410.24	1.20	370.77	1.30	–
1,4-dioxane	453.07	1.29	404.36	1.40	369.35	1.42	372.39	1.43	469.62	1.24	440.59	1.34	440.93	1.33	397.08	1.41	434
Benzene	456.04	1.31	406.74	1.41	371.27	1.43	374.35	1.44	472.73	1.25	443.45	1.35	443.79	1.35	399.37	1.42	439
Diethylether	457.02	1.30	408.90	1.40	374.18	1.42	377.12	1.43	473.26	1.25	444.73	1.34	445.06	1.34	401.52	1.42	426
Decanol	464.14	1.32	415.60	1.42	380.38	1.44	383.31	1.45	480.34	1.28	451.79	1.37	452.13	1.37	408.04	1.44	429
Dichloromethane	464.44	1.33	416.05	1.42	380.91	1.45	383.82	1.45	480.58	1.28	452.12	1.37	452.46	1.37	408.49	1.44	444.5
1-heptanol	465.41	1.33	417.09	1.43	381.95	1.45	384.84	1.45	481.48	1.28	453.10	1.37	453.45	1.37	409.51	1.44	434
1-hexanol	465.46	1.33	417.24	1.46	382.15	1.45	385.03	1.45	481.51	1.28	453.18	1.37	453.52	1.37	409.66	1.44	426.5
1-butanol	465.61	1.33	417.62	1.46	382.67	1.45	385.52	1.45	481.57	1.28	453.38	1.37	453.72	1.37	410.05	1.44	423
Acetone	464.24	1.33	416.63	1.42	381.96	1.44	384.78	1.45	480.11	1.27	452.09	1.37	452.43	1.37	409.11	1.43	436.5
Ethanol	464.70	1.33	417.10	1.42	382.42	1.44	385.23	1.45	480.54	1.28	452.54	1.37	452.89	1.37	409.57	1.43	432
Methanol	463.63	1.32	416.36	1.42	381.92	1.44	384.70	1.45	479.39	1.27	451.54	1.36	451.88	1.37	408.87	1.43	433
DMF	468.19	1.34	420.10	1.43	384.99	1.45	387.82	1.46	484.11	1.29	455.94	1.38	456.29	1.38	412.47	1.44	429.5
DMSO	467.88	1.34	419.92	1.43	384.89	1.45	387.72	1.46	483.76	1.29	455.65	1.38	456.00	1.38	412.30	1.44	452
Water	464.59	1.33	417.38	1.42	382.94	1.44	385.71	1.45	480.30	1.27	452.52	1.37	452.86	1.37	409.87	1.43	475

**Table 4:** Calculated and experimental values of excitation energies (*λ*_abs_ in nm) and oscillator strengths (f_os_) for the BS2 molecule

BS2	B3LYP		CAM-B3LYP		LC-BLYP		LC-*ω*PBE		O3LYP		PBE0		PBEh1PBE		*ω*B97XD		Exp.
	*λ*_abs_	f_os_	*λ*_abs_	f_os_	*λ*_abs_	f_os_	*λ*_abs_	f_os_	*λ*_abs_	f_os_	λ_abs_	f_os_	*λ*_abs_	f_os_	*λ*_abs_	f_os_	*λ*_abs_
Gas phase	420.33	1.21	374.95	1.33	342.69	1.35	345.78	1.37	437.01	1.14	408.38	1.25	408.66	1.25	368.77	1.35	–
1,4-dioxane	448.35	1.33	398.43	1.43	362.49	1.45	365.91	1.46	465.95	1.28	435.53	1.37	435.82	1.37	391.36	1.45	440
Benzene	451.02	1.34	400.58	1.43	364.22	1.46	367.68	1.47	468.75	1.29	438.09	1.38	438.39	1.38	393.43	1.46	437.5
Diethylether	451.70	1.33	401.99	1.43	366.11	1.45	369.47	1.46	469.18	1.28	438.97	1.38	439.26	1.37	394.77	1.45	437.5
Decanol	457.75	1.36	407.36	1.45	370.87	1.47	374.26	1.48	475.33	1.31	444.87	1.40	445.17	1.40	399.94	1.47	434
Dichloromethane	457.97	1.36	407.67	1.45	371.23	1.47	374.61	1.48	475.53	1.31	445.11	1.40	445.41	1.40	400.25	1.47	436
1-heptanol	458.80	1.36	408.48	1.45	372.01	1.47	375.38	1.48	476.34	1.31	445.94	1.40	446.23	1.40	401.03	1.47	440
1-hexanol	458.83	1.36	408.55	1.45	372.11	1.47	375.46	1.48	476.36	1.31	445.98	1.40	446.28	1.40	401.10	1.47	435
1-butanol	458.96	1.36	408.84	1.45	372.48	1.47	375.82	1.48	476.44	1.31	446.14	1.40	446.44	1.40	401.38	1.47	436
Acetone	457.74	1.35	407.93	1.45	371.82	1.47	375.12	1.48	475.14	1.30	444.98	1.40	445.28	1.39	400.51	1.46	442.5
Ethanol	458.14	1.35	408.32	1.45	372.18	1.47	375.49	1.48	475.53	1.30	445.37	1.40	445.67	1.40	400.88	1.47	434.5
Methanol	457.20	1.35	407.67	1.45	371.74	1.47	375.02	1.48	474.52	1.30	444.50	1.40	444.79	1.40	400.26	1.46	437
DMF	461.19	1.37	410.84	1.46	374.26	1.48	377.60	1.49	478.69	1.32	448.31	1.41	448.61	1.40	403.31	1.47	437.5
DMSO	460.90	1.37	410.70	1.46	374.22	1.48	377.54	1.49	478.36	1.32	448.05	1.41	448.35	1.41	403.18	1.47	465
Water	458.02	1.35	408.50	1.45	372.53	1.47	375.80	1.48	475.32	1.30	445.32	1.40	445.61	1.40	401.06	1.46	467

**Table 5:** Calculated and experimental values of excitation energies (*λ*_abs_ in nm) and oscillator strengths (f_os_) for the BS3 molecule

BS3	B3LYP		CAM-B3LYP		LC-BLYP		LC-*ω*PBE		O3LYP		PBE0		PBEh1PBE		*ω*B97XD		Exp.
	*λ*_abs_	f_os_	*λ*_abs_	f_os_	*λ*_abs_	f_os_	*λ*_abs_	f_os_	*λ*_abs_	f_os_	*λ*_abs_	f_os_	*λ*_abs_	f_os_	*λ*_abs_	f_os_	*λ*_abs_
Gas phase	424.27	1.20	378.92	1.33	346.79	1.36	349.62	1.37	440.55	1.14	412.23	1.25	412.52	1.25	372.47	1.35	–
1,4-dioxane	453.98	1.33	404.65	1.43	369.13	1.46	372.21	1.47	470.90	1.28	441.18	1.37	441.51	1.37	397.28	1.45	436.5
Benzene	456.82	1.34	406.98	1.44	371.05	1.46	374.16	1.47	473.86	1.29	443.92	1.38	444.25	1.38	399.52	1.46	438.5
Diethylether	458.18	1.34	409.47	1.44	374.24	1.46	377.22	1.47	474.81	1.29	445.56	1.39	445.88	1.38	401.97	1.46	430
Decanol	465.26	1.37	416.15	1.46	380.43	1.48	383.39	1.49	481.86	1.32	452.55	1.41	452.88	1.41	408.44	1.48	431
Dichloromethane	465.62	1.37	416.66	1.46	381.03	1.48	383.97	1.49	482.16	1.32	452.94	1.42	453.27	1.41	408.95	1.48	442
1-heptanol	466.64	1.38	417.77	1.47	382.15	1.49	385.08	1.50	483.13	1.33	453.98	1.42	454.31	1.42	410.04	1.48	434
1-hexanol	466.73	1.38	417.95	1.47	382.38	1.49	385.30	1.50	483.19	1.33	454.09	1.42	454.42	1.42	410.22	1.48	433
1-butanol	466.98	1.38	418.44	1.47	383.00	1.49	385.89	1.50	483.36	1.33	454.39	1.42	454.71	1.42	410.71	1.48	427
Acetone	465.73	1.37	417.56	1.46	382.39	1.48	385.25	1.49	482.02	1.32	453.22	1.42	453.54	1.42	409.88	1.48	433.5
ethanol	466.21	1.37	418.05	1.47	382.88	1.49	385.73	1.49	482.48	1.32	453.69	1.42	454.02	1.42	410.36	1.48	430.5
Methanol	465.25	1.37	417.41	1.46	382.48	1.48	385.30	1.49	481.45	1.32	452.81	1.42	453.13	1.41	409.76	1.48	432
DMF	469.59	1.39	420.98	1.47	385.40	1.50	388.28	1.50	485.93	1.34	456.97	1.43	457.31	1.43	413.19	1.49	430.5
DMSO	469.32	1.39	420.84	1.48	385.35	1.49	388.21	1.50	485.63	1.34	456.73	1.43	457.07	1.43	413.07	1.49	447
Water	466.26	1.38	418.49	1.47	383.55	1.49	386.36	1.49	482.41	1.32	453.82	1.42	454.15	1.42	410.82	1.48	481.5

**Fig. 4:**
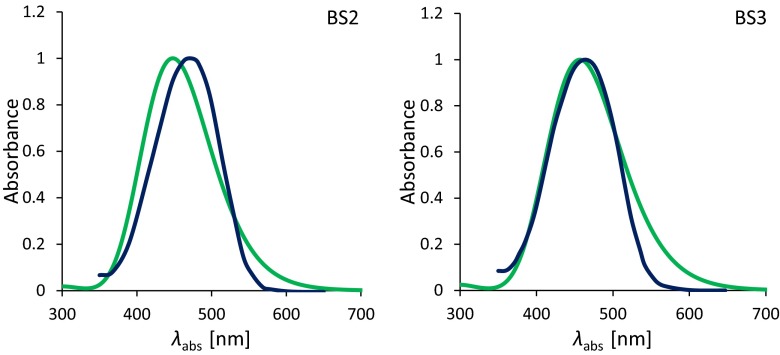
Comparison of the absorption spectra determined experimentally [67] (*blue line*) and theoretically (*green line*) based on the functional PBE0 for BS2 (*left panel*) and BS3 (*right panel*) determined in dimethylsulfoxide (DMSO). In order to reproduce the absorption band, 30 electronic transitions were taken into account. The theoretical spectra were obtained using convolutions of Gaussian distributions with a full width at half maximum of 3,387.53 cm^−1^

Figure 4 shows a comparison of the absorption spectra obtained theoretically and experimentally as determined in DMSO. In this case, two analogues of the BS2 and BS3 molecules were used, investigated earlier by Zakerhamidi et al. [67]. The difference between them lies in the replacing of the pyrimidine ring with 1,2,5-oxadiazole, leaving the residual skeleton of the compounds. The exchange in the aromatic ring, mentioned earlier, results in slight changes in the position of the maximum absorption bands. Taking into account DMSO, the *λ*_abs_ value is 465 nm for BS2 and 473 for its analogue. The difference is only about 8 nm. In the latter case, these values are 447 and 466 nm for the BS3 and its analogue, respectively. Firstly, if the calculated maximum absorption band is shifted towards shorter wavelengths, the obtained spectra for both compounds have similar shapes. In addition, for both molecules, no additional bands in the spectra obtained by measurements and theoretical calculation were detected. This indicates the absence of any specific interactions between the dissolved compound and a polar solvent and confirms the validity of the calculation methods used in determining the discussed linear and NLO properties.

As indicated by the data collected in the Tables 3 and 5, changing the solvent shifts the position of the absorption peaks. The solvatochromic shift of the maximum absorption band (Δ*ω*) can be represented by the following relationship [68]:

4 $$ \varDelta \omega =\varDelta {\omega}_E+\varDelta {\omega}_D+\varDelta {\omega}_H $$

where Δ*ω*_E_ is the pure electrostatic contribution, Δ*ω*_D_ denotes the dispersion contribution and Δ*ω*_H_ is connected with the short-range specific interaction between the solvent and dissolved molecule, e.g., hydrogen bonding. Therefore, these contributions must be also taken into account in any discussion of the spectroscopic properties of the title compounds.

TD-DFT calculations reveal that the transition of molecules from the gas phase to solution is accompanied by an increase in the OPA value (bathochromic shift). For all three azo sulfonamides this red shift is evident for weakly polar solvents (for 1-butanol). In each case, the use of a more polar acetone results in a blue shift. However, this increase in the value of the excitation energy is not reflected in the presence of other media with increasing dielectric constant. Furthermore, the value of ΔE in 1-butanol is less than that in water. It should be stressed that, in an environment characterized by a low value of relative permittivity, there is a larger polarization for the charge-transfer excited state than for the ground state. Additionally, it should be noted that, in this case, the dispersion contribution to the interaction energy between the S_2_ dissolved compound and the solvent is substantially higher than in the case of* S*_0_. On the other hand, it causes a red shift. More polar solvents, especially acetone, methanol and water, can lead to the formation of *H*-bonds involving the amino group [69–71]. This causes better stabilization of the ground state by solvation for the investigated molecules, rather than their excited state. In turn, this can induce differences that lead generally to the occurrence of negative solvatochromism (hypsochromic shift). Observations made by theoretical calculations become more valuable because if the value is measured experimentally, much larger discrepancies are obtained. For example, taking into account the transition from 1-butanol to water, experimental measurements indicate a significant shift of the maximum absorption band in the direction of longer wavelengths for each tested azo sulfonamide molecule. In addition, experimental studies indicate that the lowest value of ΔE was obtained when the medium was water, while in the case of TD-DFT calculations the environment with lowest values was DMF. In conclusion, azobenzene derivatives belong to a class of compounds with positive solvatochromism. However, in this case, the presence of the sulfamyl linker results in a negative solvatochromic shift in polar solvents. Moreover, with lowering of the solvent polarity, solvatochromic reversal occurs and the solvatochromic shift subsequently becomes positive. This phenomenon may also be induced by the tendency to self-aggregate in the low polarity solvents and in molecular structure changes from zwitterionic to neutral [72, 73]. However, these factors show that the azo sulfonamides belong to the family of dyes exhibiting solvatochromic reversal.

Table 6 illustrates the computed emission spectra λ_fluo_ in gas phase and the solvent effect on the positions of the emission peaks. The application of DFT methods for the calculation of excited states is relatively expensive. Therefore, λ_fluo_ values were determined only in three solvents of low, medium and high dielectric constants to reproduce the effect of polarity on the measured magnitude and with the use of only three DFT functionals. Moreover, photon emission is known to occur in appreciable yield only from the lowest excited state (Kasha’s rule). Therefore, the geometry optimization calculation was also conducted for the first singlet excited state (PBEO/B3LYP-6311++G(d,p)) and the λ_fluo_ values were calculated based on this geometry.

**Table 6:** Calculated and experimental values of emission spectra (*λ*_fluo_ in nm) and oscillator strengths (*f*_os_) for the title molecules

	LC-*ω*PBE			CAM-B3LYP			PBE0			Experimental
	*λ*_fluo_	f_os_	Δ*E*_2–1_^(a)^	*λ*_fluo_	f_os_	Δ*E*_2–1_^(a)^	*λ*_fluo_	f_os_	Δ*E*_2-1_^a^	*λ*_fluo_
BS1										
Gas phase	448.70	1.24		449.26	1.19		451.30	1.35		–
Acetone	460.22	1.26	0.45	468.19	1.22	0.24	500.98	1.38	0.18	537
DMSO	461.79	1.26	0.42	473.67	1.26	0.21	509.66	1.40	0.15	538
Water	461.30	1.20	0.44	472.86	1.25	0.23	508.74	1.39	0.17	543
BS2										
Gas phase	450.88	1.26		455.22	1.28		453.05	1.38		–
Acetone	463.27	1.35	0.54	471.01	1.32	0.31	503.87	1.44	0.23	534.5
DMSO	468.96	1.35	0.52	479.50	1.34	0.29	526.68	1.45	0.21	544
Water	468.11	1.33	0.53	480.17	1.33	0.30	526.06	1.42	0.22	547.5
BS3										
Gas phase	451.31	1.25		456.18	1.27		453.72	1.27		–
Acetone	458.72	1.31	0.44	473.51	1.32	0.23	502.07	1.31	0.17	534
DMSO	465.40	1.31	0.42	478.83	1.33	0.21	507.30	1.33	0.15	519
Water	465.01	1.26	0.44	478.19	1.26	0.23	507.45	1.30	0.17	547

^a^Δ*E*_2–1_ is the energy gap [eV] between the S_1_ and S_2_ state

The fluorescence can be explained by the calculated energy gap (Δ*E*_2–1_) between the* S*_2_ and* S*_1_ excited states and therefore these values are listed for all the compounds in Table 6. In each case, the calculated energy gap between S_2_ (*π* − π*) and* S*_1_ (n − π*) states is lower than 0.55 eV. The small Δ*E*_2–1_ increases the electronic coupling of the two electronically excited states, and thus facilitates an internal conversion from the* S*_2_ (bright) to* S*_1_ (dark) state. Hence, the fluorescence from the charge-transfer state cannot be measured for the investigated compounds.

**Fig. 5:**
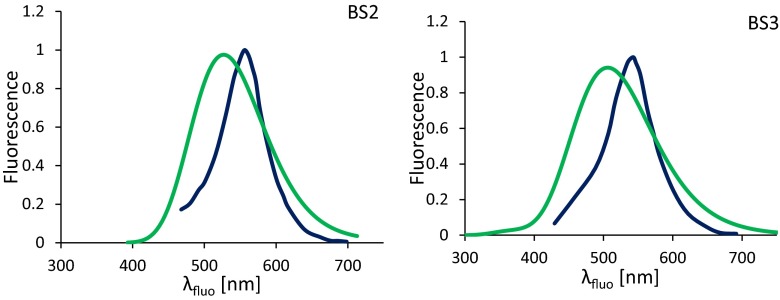
Comparison of the emission spectra determined experimentally [67] (*blue line*) and theoretically (*green line*) based on the functional PBE0 for BS2 (*left panel*) and BS3 (*right panel*) determined in DMSO. In order to reproduce the emission band, 30 electronic transitions wertr taken into account. The theoretical spectra were obtained using convolutions of Gaussian distributions with a full width at half maximum of 2984.25 cm^−1^

**Fig. 6:**
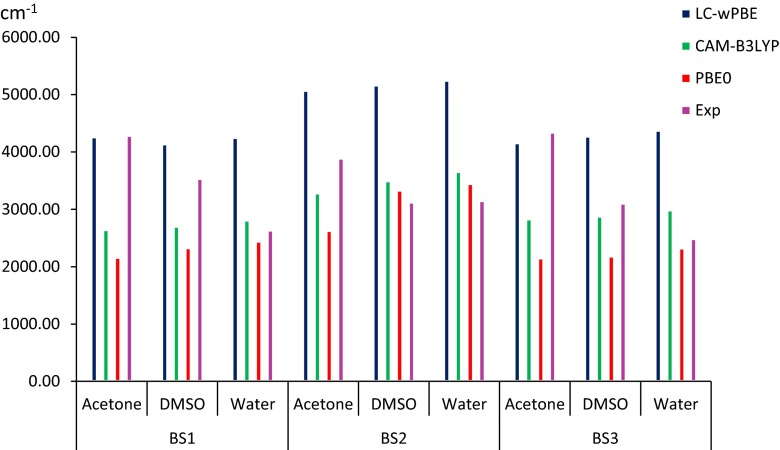
Comparison of the Stokes’ shift determined experimentally [45] with theoretical values

The data presented in Table 6 indicate that all the applied functionals underestimate the λ_fluo_ values. However, the experimental values are closest to those obtained with the participation of PBE0. The use of this functional leads to a shift in the λ_fluo_ towards shorter wavelengths relative to the measured one, and the average difference Δ*λ*_exp - PBE0_ between them is 41, 36 and 33 nm for BS1, BS2 and BS3, respectively. Additionally, the theoretical peaks have similar intensities and shape (see Fig. 5). In turn, the LC functionals gives fluorescence values at a similar level but generally lower when comparing to PBE0. The differences with respect to the experimental values are as high as 70 nm for the LC-*ω*PBE and 60 nm for the CAM-B3LYP. Taking into account the value of the Stokes’ shift (Δ*ν*), values closest to the experimental values are obtained using asymptotically corrected functionals. As seen in Fig. 6, the LC-*ω*PBE correctly describes Δ*ν* in the presence of an environment characterized by a low value of the relative permittivity, while CAM-B3LYP describes them correctly in more polar solvents. This better consistency for the long-range corrected functionals in relation to experiment findings is due mainly to the fact that they yield differences of maximal absorption and emission bands (Δ*λ*_fluo_ − *λ*_abs_) values closer to the measured ones than does PBE0.

Theoretical values (Table 6) show that the transition from the vacuum to the solution is accompanied by a red shift. This bathochromic shift occurs up to DMSO; passing further towards water results in a hypsochromic shift. This negative solvatochromism is not evident in the available experimental data. However, analysis of the values obtained by Zakerhamidi et al. [45] indicates a different solvatochromic change when changing the polarity of the medium. For example, for decanol, the following values of λ_fluo_ were recorded: 580, 583 and 579.5 nm for BS1, BS2 and BS3, respectively. In turn, for ethanol these values were 520, 528 and 553 nm. In this context, the influences in the emission spectra of polar prototic and aprototic solvents with H-bond properties are also highlighted. More importantly, these relationships are properly described by DFT methods. On the other hand, this observation, which is in accordance with the conclusions from the OPA, confirms that values of the relative electric permittivity of the environment (the electrostatic contribution), the dispersion contribution and *H*-bonds between azo sulfonamide dyes and solvent molecules make an effective contribution to ground and excited state stabilization and thus influence the spectroscopic parameters. More importantly, these observations are reproduced in the geometrical parameters mentioned previously. From the data presented in Tables 1 and 2, it is clear that the causes of this phenomenon can be discerned in the distance at which the sulfamyl linker is located relative to neighboring pyrimidine ring and azobenzene. On the other hand, the size of the solvatochromic shift is also affected by the presence of the π-bridge. In this case, the bond lengths between atoms, but not the angle at which they are arranged, have a decisive influence on the presence of the red or blue shift in the absorption and fluorescence spectra or on the contributions to ground and excited state stabilization.

### Polarity of low-lying excited state

The calculated dipole moments for the ground (*μ*_0_) and the second excited (*μ*_2_) states for the investigated systems are listed in Table 7. It can be seen that the differences in the values of dipole moments obtained using different functionals are not significant. However, both B3LYP and PBE0 predict similar values of dipole moments and the difference does not exceed 0.23 and 0.16 D for the ground and second singlet excited states, respectively. On the other hand, the B3LYP gives higher values for* S*_0_ relative to PBE0, whereas the second functional leads to a higher value of* S*_2_. Referring to the PBE0, the CAM-B3LYP estimated value of the *μ*_0_ and *μ*_2_ is lower by about 0.15 D and 1 D, respectively. On the other hand, taking into account LC functionals, they give almost the same values of the dipole moments. It should be mentioned that, with respect to the LC-B3LYP, LC-*ω*PBE gives lower values of* S*_0_ by about 0.06 D for BS1 and 0.02 D for BS3, and higher by about 0.3 D for BS2. In the case of a charge transfer state, LC-*ω*PBE provides lower values by about 0.03–0.05 and 0.3–0.15 D higher with respect to LC-B3LYP, for BS1 and BS2, respectively. For the BS3 molecule, the first functional leads to higher values of 0.02–0.03 D, with the exception of diethylether, which gave a value of *μ*_2_ higher by as much as 0.9 D. In general, the differences do not exceed 2 and 1.65 D with respect to the PBE0, for* S*_0_ and* S*_2_, respectively. More importantly, for each tested compound, dipole moments over 8 D higher in the excited state (*μ*_0_ < *μ*_2_) were obtained, which indicates a significant polarity of the CT state (Δ*μ*_g-CT_). Furthermore, in each case, the lowest values of dipole moments were obtained in the gas phase. The transition to higher electric permittivity of the environment was accompanied by a gradual increase in both *μ*_0_ and *μ*_2_. Therefore, this correlation is not in accordance with the transition energies, ΔE. On the other hand, for molecules with larger polarization for the charge-transfer excited state than for the ground state, the presence of a red shift is characteristic [68].

**Table 7:** Theoretical values of dipole moments for the ground and second lowest-lying singlet excited states calculated at the TDDFT/6-311++nd,p) level of theory. All values are given in D

	B3LYP		CAM-B3LYP		LC-BLYP		LC-*ω*PBE		PBE0	
	*μ*_0_	*μ*_2_	*μ*_0_	*μ*_2_	*μ*_0_	*μ*_2_	*μ*_0_	*μ*_2_	*μ*_0_	*n*_2_
BS1										
Gas phase	11.27	18.00	10.58	17.37	10.13	17.20	10.07	17.23	11.16	18.15
1.4-dioxane	13.11	20.93	12.70	20.29	11.56	20.19	11.50	20.19	12.96	21.08
Benzene	13.15	21.05	12.21	20.42	11.59	20.32	11.53	20.32	12.99	21.20
Diethylether	14.16	22.30	13.07	21.62	12.36	21.53	12.30	21.51	13.99	22.45
Decanol	14.79	23.15	13.61	22.47	12.84	22.42	12.78	22.39	14.60	23.29
Dichloromethane	14.93	23.31	13.73	22.62	12.94	22.58	12.89	22.55	14.73	23.45
1-heptanol	15.09	23.50	13.87	22.82	13.06	22.79	13.01	22.75	14.89	23.65
1-hexanol	15.15	23.57	13.92	22.89	13.11	22.85	13.05	22.81	14.95	23.72
1-butanol	15.32	23.75	14.06	23.06	13.23	23.04	13.18	22.99	15.11	23.90
Acetone	15.38	23.80	14.11	23.10	13.28	23.07	13.23	23.03	15.18	23.94
Ethanol	15.45	23.88	14.17	23.18	13.33	23.15	13.28	23.11	15.24	24.02
Methanol	15.52	23.94	14.23	23.23	13.39	23.20	13.33	23.16	15.31	24.08
DMF	15.55	24.05	14.26	23.37	13.41	23.36	13.36	23.31	15.34	24.20
DMSO	15.59	24.09	14.29	23.40	13.44	23.39	13.39	23.35	15.38	24.24
Water	15.67	24.15	14.35	23.46	13.50	23.46	13.44	23.36	15.45	24.29
BS2										
Gas phase	10.14	17.19	9.37	16.35	8.83	15.95	8.85	16.11	10.03	17.35
1.4-dioxane	11.59	19.80	10.60	18.89	9.90	18.48	9.93	18.63	11.44	19.95
Benzene	11.63	19.90	10.63	19.00	9.93	18.59	9.96	18.74	11.48	20.05
Diethylether	12.43	21.02	11.31	20.03	10.53	19.61	10.56	19.75	12.26	21.16
Decanol	12.99	21.85	11.78	20.83	10.95	20.41	10.98	20.55	12.79	21.98
Dichloromethane	13.11	22.01	11.89	20.98	11.04	20.55	11.08	20.69	12.91	22.14
1-heptanol	13.24	22.19	11.99	21.15	11.14	20.73	11.17	20.87	13.04	22.32
1-hexanol	13.29	22.25	12.04	21.21	11.17	20.79	11.21	20.92	13.09	22.38
1-butanol	13.42	22.42	12.15	21.36	11.27	20.94	11.31	21.08	13.22	22.55
Acetone	13.47	22.46	12.19	21.39	11.31	20.97	11.34	21.10	13.26	22.59
Ethanol	13.52	22.53	12.23	21.46	11.34	21.04	11.38	21.17	13.31	22.66
Methanol	13.58	22.59	12.28	21.50	11.39	21.08	11.42	21.21	13.37	22.71
DMF	13.60	22.69	12.30	21.63	11.40	21.19	11.44	21.34	13.39	22.80
DMSO	13.63	22.73	12.32	21.65	11.42	21.22	11.46	21.36	13.42	22.84
Water	13.68	22.79	12.36	21.71	11.46	21.27	11.49	21.39	13.47	22.89
BS3										
Gas phase	9.48	16.42	8.77	15.74	8.32	15.52	8.28	15.59	9.35	16.53
1.4-dioxane	11.02	18.99	10.08	18.29	9.47	18.13	9.44	18.18	10.85	19.12
Benzene	11.07	19.15	10.12	18.40	9.50	18.25	9.48	18.30	10.89	19.22
Diethylether	11.97	20.21	10.88	19.46	10.17	19.32	10.15	20.24	11.77	20.33
Decanol	12.56	21.01	11.38	20.27	10.61	20.15	10.59	20.18	12.35	21.14
Dichloromethane	12.68	21.16	11.48	20.41	10.70	20.30	10.68	20.33	12.47	21.29
1-heptanol	12.84	21.34	11.61	20.59	10.81	20.49	10.79	20.51	12.62	21.47
1-hexanol	12.89	21.4	11.66	20.64	10.85	20.55	10.84	20.57	12.67	21.53
1-butanol	13.04	21.57	11.78	20.80	10.97	20.71	10.95	20.74	12.82	21.69
Acetone	13.11	21.61	11.84	20.84	11.01	20.75	10.99	20.77	12.88	21.73
Ethanol	13.17	21.68	11.89	20.91	11.06	20.82	11.04	20.84	12.94	21.80
Methanol	13.24	21.74	11.95	20.95	11.11	20.87	11.09	20.89	13.01	21.94
DMF	13.26	21.81	11.97	21.09	11.13	21.01	11.11	21.03	13.03	21.97
DMSO	13.31	21.86	12.00	21.12	11.16	21.05	11.14	21.07	13.07	22.07
Water	13.37	21.93	12.06	21.19	11.21	21.12	11.19	21.13	13.14	22.15

**Fig. 7:**
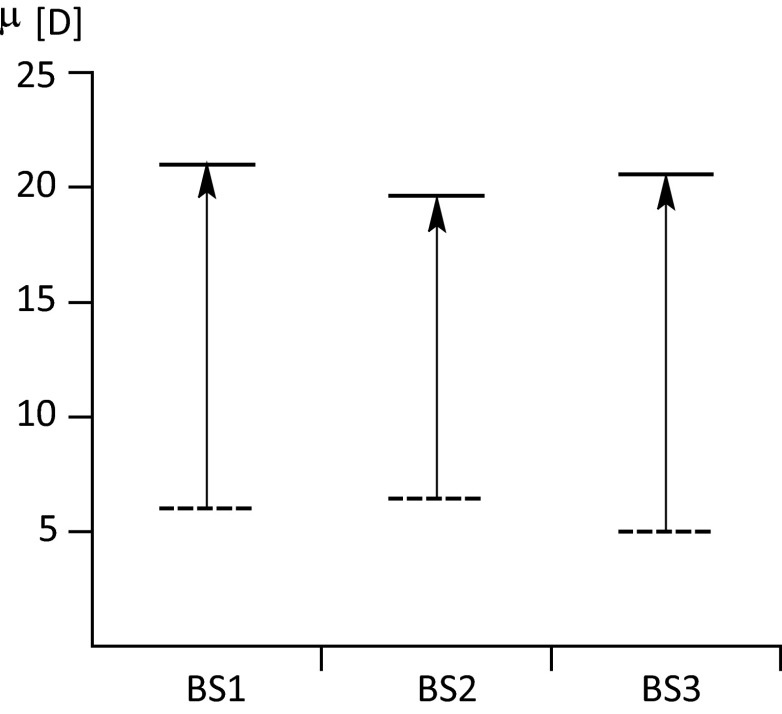
Experimental values of dipole moments in the ground (*dotted line*) and low-lying excited (*solid line*) state obtained by Zakerhamidi et al. [45]

It is difficult to compare the *μ* values determined experimentally with calculated ones. To estimate dipole moments, the Zakerhamidi group [45] used illustrations of spectral shifts for the solute compounds as a function of solvent polarity. Next, using a linear curve fitting approach, the m_1_ and m_2_ parameters were found, and the ground and the excited dipole moments were calculated on this basis. The result of these approximations is shown in Fig. 7. It can be seen that the DFT functionals lead to slightly higher values of *μ*_0_ relative to experimental values, with almost identical values reproduced for *μ*_2_. Moreover, the values calculated indicate that the BS1 molecule is characterized by the highest values of both* μ*_0_ and *μ*_2_:

$$ {\mu}_0\ /{\mu}_{2\ }\mathrm{B}\mathrm{S}1>{\mu}_0\ /{\mu}_{2\ }\mathrm{B}\mathrm{S}2>{\mu}_0\ /{\mu}_{2\ }\mathrm{B}\mathrm{S}3 $$

The experimental values do not classify the tested azo sulfonamides in the same way:

$$ \begin{array}{c}\hfill {\mu}_{0\ }\mathrm{B}\mathrm{S}2>{\mu}_{0\ }\mathrm{B}\mathrm{S}1>{\mu}_{0\ }\mathrm{B}\mathrm{S}3\hfill \\ {}\hfill {\mu}_{2\ }\mathrm{B}\mathrm{S}1>{\mu}_{2\ }\mathrm{B}\mathrm{S}3>{\mu}_{2\ }\mathrm{B}\mathrm{S}2\hfill \end{array} $$

This may result from the effect of the *H*-bonding solute–solvent interactions on the measured excitation energy. Thus, in this case, the calculated values may be more correct. It is worth noting that the dipole moment values are also determined by the nature of the D/A substituents. The azo sulfonamides discussed here differ in structure in their amino group, which plays the electron-donating role. As previously shown, a rise in the strength of the donor group results in increased *μ*_0_ and *μ*_2_ values when the acceptor stays the same [74]. The dimethylamino group is a substituent of medium strength. According to the data presented, its strength is weakened by the presence of an oxygen atom with electron-accepting properties. Furthermore, the farther the oxygen is from the nitrogen atom, the lower the strength of the dimethylamino group, hence the classification of the title compounds as above.

The results show that the tested azo sulfonamides, as a group of the D-*π*-A chromophores, are characterized by the existence of excited states with large differences in polarity between the electronic excited state and the ground state (Δ*μ*_g-CT_). This is one of the parameters that determines the NLO activity of molecular systems. The Δ*μ*_g-CT_ calculated values show that the higher dielectric constant of the solvent results in an increase in polarity of the charge-transfer state; see Table S4 in the Supporting Information. Although the ΔΔ*μ*_g-CT_ becomes smaller in more polar solvents, the title compounds are suggested to have a positive solvatochromism. On the other hand, the use of different DFT functionals does not result in such large differences in the obtained Δ*μ*_g-CT_, as was the case with ΔE. However, the following relationship for the dipole moment difference between the ground and the excited state was found:

$$ \varDelta {\mu}_{\mathrm{g} - \mathrm{C}\mathrm{T}}^{\mathrm{LC}}>\varDelta {\mu}_{\mathrm{g} - \mathrm{C}\mathrm{T}}^{\mathrm{CAM}-\mathrm{B}3\mathrm{L}\mathrm{Y}\mathrm{P}} > \varDelta {\mu}_{\mathrm{g} - \mathrm{C}\mathrm{T}}^{\mathrm{PBE}0}>\varDelta {\mu}_{\mathrm{g} - \mathrm{C}\mathrm{T}}^{\mathrm{B}3\mathrm{L}\mathrm{Y}\mathrm{P}} $$

More importantly, these values are lower than those determined experimentally. In the case of BS1, the ∆μ_g-CT_^Exp^ – ∆μ_g-CT_^theoretical^ is about 6 D, 4 D for the BS2 and 6.5 D for the BS3. Not only does the difference in the obtained values become significant, but also the classification of tested compounds. Analysis of the polarity of the CT excited state gives the following order:

$$ \varDelta {\mu}_{\mathrm{g}-\mathrm{C}\mathrm{T}}\mathrm{B}\mathrm{S}2>\varDelta {\mu}_{\mathrm{g}-\mathrm{C}\mathrm{T}}\mathrm{B}\mathrm{S}1>\varDelta {\mu}_{\mathrm{g}-\mathrm{C}\mathrm{T}}\mathrm{B}\mathrm{S}3 $$

for the calculated and

$$ \varDelta {\mu}_{\mathrm{g}-\mathrm{C}\mathrm{T}}\mathrm{B}\mathrm{S}3>\varDelta {\upmu}_{\mathrm{g}-\mathrm{C}\mathrm{T}}\mathrm{B}\mathrm{S}1>\varDelta {\mu}_{\mathrm{g}-\mathrm{C}\mathrm{T}}\mathrm{B}\mathrm{S}2 $$

for the experimentally determined values. The reasons for these discrepancies should be seen in the absence of the dipole moments determined experimentally in environments of different polarity. Therefore, the theoretical values are complementary to the measured values. Nevertheless, both calculation and experiment show the high polarity of the charge-transfer excited state of the azo sulfonamides, which is characteristic of the red shift. In addition, the connection of Δ*μ*_g-CT_ with the excitation energies suggests that the compounds analyzed should be characterized by a high value of the non-linear response, like the two-photon absorption cross section.

### Two-photon absorption cross section

For compounds with positive solvatochromism, the two-photon absorption spectrum shifts toward longer wavelengths with increasing polarity of the solvent [68, 70, 71, 74]. All the results presented here indicate that the studied azo sulfonamide dyes are characterized by a reversal solvatochromism. Unfortunately, no experimental measurements or information on the impact of environments with different polarity on 2PA values for the tested azo sulfonamide dyes are currently available. For this reason the values presented below should be treated as demonstrative and will be compared to those of azobenzene derivatives [74].

Theoretically determined values of 2PA cross section in a.u. 〈*δ*^OF^〉 and in GM (*σ*_OF_^(2)^) for the compounds tested in this study are given in Table 8 for the lowest singlet excited state. The calculations were performed using two DFT functionals. The CAM-B3LYP values of 〈*δ*^OF^〉 are much smaller than those calculated using asymptotically incorrect functionals, namely B3LYP. In general, 〈*δ*^OF^〉 values are significantly larger in the presence of a solvent. The increase can be as large as 150 %. Moreover, the values of 〈*δ*^OF^〉 increase with increasing polarity of the environment. This is consistent with the observations made for the polarity of the excited state. It is not surprising that 〈*δ*^OF^〉 is quite sensitive to the nature of the D/A substituents. As in the case of the *μ*_0_ / *μ*_2_, in eachmedium the highest values of 〈*δ*^OF^〉 were obtained for BT1. For this compound, these values are higher by more than 15,000 a.u. for the B3LYP and more than 8000 a.u. for the CAM-B3LYP relative to BS1 and BS2. The BS2 and BS3 molecules are characterized by similar values of 〈*δ*^OF^〉. However, for the former the obtained values are higher by about 3500 and 2000 a.u. for the B3LYP and CAM-B3LYP, respectively. Therefore, the ordering of the analyzed azo sulfonamide dyes is predicted as:

$$ {\delta}^{\mathrm{OF}}BS1>{\delta}^{\mathrm{OF}}BS2>{\delta}^{\mathrm{OF}}BS3 $$

**Table 8:** Results of calculations of two-photon absorption (2PA) cross-section. The values of 〈*δ* ^OF^〉 ^×^10^−3^ are given in a.u. and (σ_OF_^(2)^) in GM

	〈*δ* ^OF^〉		(σ_OF_^(2)^)	
	B3LYP	CAM-B3LYP	B3LYP	CAM-B3LYP
BS1				
Gas phase	27.6	22.9	69.5	72.5
1-hexanol	102.2	58.9	260.5	186.7
Acetone	102.3	59.3	262.5	189.1
Methanol	105.1	60.5	269.7	192.9
DMSO	121.3	63.1	311.2	202.5
Water	121.7	63.9	308.1	203.8
BS2				
Gas phase	25.2	21.4	63.8	67.7
1-hexanol	86.2	50.3	219.7	157.5
Acetone	86.6	50.3	220.7	158.5
Methanol	86.7	50.4	219.5	158.8
DMSO	88.9	51.7	228.1	164.1
Water	89.0	52.1	226.8	164.1
BS3				
Gas phase	23.6	19.9	59.8	63.3
1-hexanol	82.8	48.2	208.2	152.8
Acetone	83.0	48.4	210.1	153.4
Methanol	83.0	48.5	210.1	154.7
DMSO	84.4	49.1	215.2	157.5
Water	84.6	49.3	212.7	157.2

The presence of a strong electron-donating substituent (the dimethylamino group) enhances the values of the two-photon cross section. From the viewpoint of two-photon imaging applications, it becomes pointless to increase the length of the alkyl chains with oxygen atoms in the amino group. On the one hand, this weakens the strength of electron-donating, and on the other, it leads to a decrease in 〈*δ*^OF^〉 value by a factor of 1.5. This conclusion is consistent with experimental and theoretical studies carried out for the azobenzene derivatives [57, 74].

In order to compare calculated values with those measured experimentally (*σ*_OF_^(2)^), the 2PA cross-section must be expressed in GM units (see Eq. 2). Due to the absence of experimental values, in all simulations *Γ*_F_ was assumed to be equal to 0.25 eV [74, 75]. Looking closely at Table 8, it can be seen that a change in polarity of the solvent is accompanied by an increase in the (*σ*_OF_^(2)^) value. More importantly, the two-photon absorptivities tend to increase with the polarity of the environment, but only up to DMSO. The transition from DMSO to the more polar water results in a reduction in the (*σ*_OF_^(2)^) values. This relationship differs from those obtained for the dipole moments, but is consistent with the absorption and emission spectra. As presented previously [68, 71, 74–76], the two-photon cross section depends on three parameters: the transition energy, the oscillator strength and the polarity of the excited state. According to the data provided, f_OS_ and Δ*μ*_g-CT_ are high and almost equal in all solvents. Therefore, the ΔE has a decisive influence on the 2PA cross-section. Consequently, the quantity discussed in terms of solvent impact on the obtained values results inconclusions analogous to those in the case of the one-photon absorption. For this reason, reversed solvatochromism was observed in the 2PA spectra of the investigated azo sulfonamide. Secondly, the dependence of the effect of the donor substituent on the obtainable value is consistent with 〈*δ*^OF^〉 values. Moreover, and even more importantly for critical assessment of the applied models, the CAM-B3LYP functional leads to a smaller value; however, in accordance with previous studies and comparisons with measured values [70, 71, 74], it gives the most reliable results, which are in good agreement with experimental values.

## Conclusions

Density functional theory calculations were carried out to study the structure, as well as the linear and NLO properties, of three azo sulfonamide dyes. The calculated results show that, during the excitation, structural changes occur in the investigated compounds. These changes are mainly in the sulfamyl linker, NN π-bridge and amino group. In the excited state, the sulfamyl linker bonds become shorter and the whole fragment moves in the direction of the pyrimidine ring. In contrast, the NN π-bridge moves in accordance with the charge transfer, which results in moving away from the ring with the amino group in the direction of the ring with the attached sulfone group. In turn, in the amino group only the first methyl groups connected to the nitrogen atom are changed. The presence of a weakly polar solvent enhances these changes. In addition, these studies have shown that the pyrimidine ring is not involved in intramolecular charge transfer, although it has donating groups; upon excitation, only twisting of this ring occurs. The value of the energy separation between the HOMO and LUMO is very large and indicates high chemical hardness for the investigated compounds. The results obtained during TD-DFT calculations are in good agreement with the experimental absorption and emission spectra and the most reliable values are obtained for the PBE0 functional. The calculations show that, although the transition from the gas phase into the poorly polar solvent is accompanied by a bathochromic shift, the titled compounds are characterized by the presence of reversible solvatochromism. More polar environments can lead to the formation of H-bonds, which result in better stabilization of the ground state than the excited state. Moreover, the presence of a sulfamyl linker results in a negative solvatochromic shift in some of the polar environments. These phenomena suggest that, depending on the polarity and proticity of the medium, the better stabilization occurs in the case of one of two valance-bond forms, namely neutral and zwitterionic. Theoretical calculations showed that the energy gaps between the* S*_2_ and* S*_1_ states for all investigated molecules are smaller than 0.5 eV, which facilitates nonradiative deactivation of the charge-transfer excited state, and thus* S*_2_ fluorescence is quenched. The results show that all these compounds are characterized by a high polarity of the charge-transfer excited state. Despite the fact that this property is characteristic of the occurrence of positive solvatochromism, it indicates that the ground state of the neutral geometry is stabilized more than the zwitterionic structure, and that self-aggregation of azo sulfonamide dyes should not occur in consideration solvents. On the other hand, the theoretical calculations on the investigated D-π-A chromophores predicted non-monotonic behavior of the 2PA cross section with increasing solvent polarity. It was found that the computed 2PA cross section was smaller in water solution than in DMSO. Although an accurate estimation of the width of the absorption band, *Γ*_F_, is crucial for qualitative predictions of the 2PA cross sections; the values obtained clearly confirm that the compounds analyzed are characterized by reversible solvatochromism. In general, this type of donor-acceptor π-conjugated molecule is promising for applications in various linear and NLO devices. A large Stokes’ shift value and the 2PA cross section makes these compounds useful in bioimaging.

## Electronic supplementary material

ESM 1 (DOCX 55 kb)
