# Supplementary material for: Time-dependent density functional theory calculations of the solvatochromism of some azo sulfonamide fluorochromes
Source: J Mol Model. 2015 Apr 16;21(5):118. doi: 10.1007/s00894-015-2651-z (PMC4427650; doi:10.1007/s00894-015-2651-z)
Supplement: Supplementary file 1 — (DOCX 55 kb) [file 894_2015_2651_MOESM1_ESM.docx]

**SUPPORTING INFORMATION**

**Time-Dependent Density Functional Theory Calculations of the Solvatochromism of Some Azo Sulfonamide Fluorochromes**

Przemysław Krawczyk*

Nicolaus Copernicus University, Collegium Medicum, Department of Physical Chemistry

Kurpińskiego 5, 85-950 Bydgoszcz, Poland

**TABLES**

**Table S1** Structural parameters in the ground (S_0_) and the charge-transfer excited state (S_2_) of the BS1 compound determined at the time-dependent functional theory PBE0/6-311++G(d,p) level of theory with the inclusion of solvent effects. The bond lengths are given in (Å) and bond and torsion angles in (^o^)

|  | gas phase | | acetone | | DMSO | | water | |
| --- | --- | --- | --- | --- | --- | --- | --- | --- |
|  | S_0_ | S_2_ | S_0_ | S_2_ | S_0_ | S_2_ | S_0_ | S_2_ |
| bond lengths |  |  |  |  |  |  |  |  |
| 2-3 | 1.503 | 1.497 | 1.502 | 1.495 | 1.502 | 1.496 | 1.502 | 1.497 |
| 2-4 | 1.347 | 1.346 | 1.348 | 1.345 | 1.348 | 1.344 | 1.348 | 1.345 |
| 4-5 | 1.333 | 1.332 | 1.335 | 1.332 | 1.335 | 1.332 | 1.335 | 1.332 |
| 5-6 | 1.503 | 1.499 | 1.503 | 1.496 | 1.503 | 1.496 | 1.503 | 1.498 |
| 5-7 | 1.341 | 1.340 | 1.342 | 1.337 | 1.342 | 1.336 | 1.342 | 1.338 |
| 7-8 | 1.333 | 1.331 | 1.334 | 1.334 | 1.334 | 1.334 | 1.334 | 1.333 |
| 8-9 | 1.407 | 1.398 | 1.406 | 1.393 | 1.406 | 1.395 | 1.406 | 1.396 |
| 9-10 | 1.715 | 1.699 | 1.708 | 1.695 | 1.707 | 1.697 | 1.707 | 1.698 |
| 10-11 | 1.461 | 1.459 | 1.466 | 1.466 | 1.466 | 1.465 | 1.466 | 1.464 |
| 10-12 | 1.460 | 1.458 | 1.465 | 1.464 | 1.465 | 1.464 | 1.465 | 1.464 |
| 10-13 | 1.789 | 1.754 | 1.782 | 1.745 | 1.782 | 1.740 | 1.782 | 1.743 |
| 16-19 | 1.416 | 1.344 | 1.412 | 1.341 | 1.412 | 1.339 | 1.412 | 1.345 |
| 19-20 | 1.261 | 1.247 | 1.267 | 1.247 | 1.268 | 1.248 | 1.268 | 1.249 |
| 20-21 | 1.396 | 1.351 | 1.387 | 1.351 | 1.387 | 1.354 | 1.386 | 1.377 |
| 24-27 | 1.372 | 1.373 | 1.361 | 1.360 | 1.361 | 1.362 | 1.360 | 1.361 |
| 27-28 | 1.456 | 1.443 | 1.461 | 1.444 | 1.461 | 1.447 | 1.461 | 1.448 |
| 27-29 | 1.457 | 1.444 | 1.462 | 1.343 | 1.462 | 1.449 | 1.462 | 1.447 |
| bond angles |  |  |  |  |  |  |  |  |
| 8-9-10 | 124.139 | 124.517 | 124.016 | 124.385 | 124.119 | 124.414 | 124.166 | 124.424 |
| 9-10-13 | 99.550 | 99.986 | 100.230 | 101.078 | 100.284 | 100.843 | 100.302 | 100.749 |
| 1-8-9 | 124.098 | 123.876 | 124.027 | 123.708 | 124.032 | 123.781 | 124.034 | 123.838 |
| 7-8-9 | 113.557 | 113.305 | 113.708 | 113.831 | 113.707 | 113.793 | 113.705 | 113.745 |
| 9-10-11 | 110.314 | 110.500 | 110.597 | 109.652 | 110.615 | 109.766 | 110.620 | 109.987 |
| 9-10-12 | 106.400 | 106.671 | 106.504 | 105.306 | 106.513 | 105.369 | 106.515 | 105.394 |
| 10-13-14 | 119.025 | 119.285 | 119.154 | 119.768 | 119.166 | 119.723 | 119.170 | 119.537 |
| 10-13-18 | 119.370 | 119.605 | 119.212 | 119.622 | 119.198 | 119.501 | 119.194 | 119.340 |
| 15-16-19 | 124.712 | 124.451 | 124.893 | 121.935 | 124.903 | 122.299 | 124.907 | 123.940 |
| 17-16-19 | 115.493 | 118.395 | 115.530 | 118.626 | 115.530 | 118.359 | 115.531 | 117.200 |
| 16-19-20 | 114.591 | 130.833 | 114.613 | 132.505 | 114.620 | 132.187 | 114.622 | 130.907 |
| 19-20-21 | 116.284 | 130.18 | 116.690 | 131.129 | 116.95 | 131.207 | 116.99 | 131.246 |
| 20-21-22 | 116.311 | 118.645 | 116.324 | 118.297 | 116.326 | 118.254 | 116.326 | 117.247 |
| 20-21-26 | 125.296 | 124.080 | 125.510 | 122.701 | 125.521 | 123.216 | 125.525 | 124.543 |
| 23-24-27 | 121.383 | 121.437 | 120.392 | 120.882 | 121.480 | 120.882 | 121.482 | 120.904 |
| 25-24-27 | 121.084 | 121.413 | 120.692 | 121.106 | 121.104 | 121.118 | 121.104 | 121.236 |
| 24-27-28 | 120.229 | 120.044 | 120.392 | 120.349 | 120.400 | 120.411 | 120.402 | 120.531 |
| 24-27-29 | 120.514 | 120.161 | 120.692 | 120.081 | 120.701 | 120.332 | 120.704 | 120.388 |
| 28-27-29 | 119.231 | 119.789 | 118.910 | 119.568 | 118.894 | 119.655 | 118.888 | 119.674 |
| torsion angles |  |  |  |  |  |  |  |  |
| 1-8-9-10 | 35.635 | 33.549 | 34.004 | 33.505 | 33.940 | 33.251 | 33.916 | 33.018 |
| 7-8-9-10 | -146.924 | -148.781 | -148.465 | -149.438 | -148.531 | -149.399 | -148.555 | -149.288 |
| 8-9-10-11 | 76.424 | 73.277 | 71.868 | 70.717 | 71.771 | 70.645 | 71.755 | 70.583 |
| 8-9-10-12 | -55.286 | -54.24 | -57.867 | -56.802 | -58.872 | -56.996 | -59.857 | -57.526 |
| 8-9-10-13 | -169.857 | -173.030 | -174.906 | -179.666 | -173.204 | -175.138 | -173.197 | -174.200 |
| 9-10-13-14 | -105.097 | -106.791 | -105.842 | -107.265 | -105.845 | -107.224 | -105.843 | -106.760 |
| 9-10-13-18 | 75.752 | 74.552 | 74.897 | 73.743 | 74.910 | 73.773 | 74.918 | 74.108 |
| 15-16-19-20 | 0.123 | 0.030 | 0.353 | 1.473 | 0.485 | 1.650 | 0.524 | 1.986 |
| 17-16-19-20 | 179.662 | 179.748 | -179.745 | -178.842 | -179.612 | 178.788 | -179.571 | 178.744 |
| 16-19-20-21 | 179.946 | 177.855 | 179.176 | 176.494 | 179.901 | 176.620 | 179.901 | 176.636 |
| 19-20-21-22 | 179.948 | -179.866 | -179.919 | -178.537 | -179.869 | -177.975 | -179.852 | -177.081 |
| 19-20-21-26 | 0.141 | 0.098 | 0.033 | 1.659 | 0.033 | 2.016 | 0.016 | 2.308 |
| 23-24-27-28 | 0.846 | 0.168 | 0.214 | 0.311 | 0.247 | 0.544 | 0.261 | 0.656 |
| 25-24-27-29 | -1.013 | -0.709 | -0.653 | -0.003 | -0.625 | -0.115 | -0.617 | -0.449 |

**Table S2** Structural parameters in the ground (S_0_) and the charge-transfer excited state (S_2_) of the BS2 compound determined at the time-dependent functional theory PBE0/6-311++G(d,p) level of theory with the inclusion of solvent effects**.** The bond lengths are given in (Å) and bond and torsion angles in (^o^)

|  | gas phase | | acetone | | DMSO | | water | |
| --- | --- | --- | --- | --- | --- | --- | --- | --- |
|  | S_0_ | S_2_ | S_0_ | S_2_ | S_0_ | S_2_ | S_0_ | S_2_ |
| bond lengths |  |  |  |  |  |  |  |  |
| 2-3 | 1.503 | 1.498 | 1.502 | 1.497 | 1.502 | 1.493 | 1.502 | 1.496 |
| 2-4 | 1.346 | 1.344 | 1.349 | 1.346 | 1.348 | 1.346 | 1.348 | 1.345 |
| 4-5 | 1.333 | 1.332 | 1.335 | 1.332 | 1.335 | 1.332 | 1.335 | 1.333 |
| 5-6 | 1.503 | 1.498 | 1.503 | 1.498 | 1.503 | 1.498 | 1.503 | 1.498 |
| 5-7 | 1.341 | 1.337 | 1.342 | 1.338 | 1.342 | 1.338 | 1.342 | 1.338 |
| 7-8 | 1.332 | 1.333 | 1.333 | 1.333 | 1.334 | 1.333 | 1.334 | 1.333 |
| 8-9 | 1.408 | 1.388 | 1.407 | 1.395 | 1.406 | 1.392 | 1.406 | 1.393 |
| 9-10 | 1.714 | 1.693 | 1.708 | 1.695 | 1.707 | 1.703 | 1.707 | 1.704 |
| 10-11 | 1.461 | 1.454 | 1.466 | 1.473 | 1.466 | 1.491 | 1.466 | 1.469 |
| 10-12 | 1.460 | 1.455 | 1.465 | 1.463 | 1.465 | 1.463 | 1.465 | 1.463 |
| 10-13 | 1.790 | 1.755 | 1.783 | 1.742 | 1.783 | 1.737 | 1.783 | 1.742 |
| 16-19 | 1.416 | 1.365 | 1.414 | 1.342 | 1.414 | 1.341 | 1.415 | 1.343 |
| 19-20 | 1.260 | 1.303 | 1.265 | 1.244 | 1.266 | 1.246 | 1.266 | 1.248 |
| 20-21 | 1.397 | 1.402 | 1.390 | 1.354 | 1.389 | 1.356 | 1.389 | 1.357 |
| 24-27 | 1.382 | 1.382 | 1.373 | 1.375 | 1.373 | 1.375 | 1.373 | 1.375 |
| 27-28 | 1.461 | 1.447 | 1.464 | 1.450 | 1.464 | 1.449 | 1.464 | 1.449 |
| 27-29 | 1.461 | 1.446 | 1.464 | 1.450 | 1.465 | 1.443 | 1.465 | 1.440 |
| 30-31 | 1.425 | 1.406 | 1.430 | 1.411 | 1.430 | 1.404 | 1.430 | 1.402 |
| 32-33 | 1.425 | 1.406 | 1.430 | 1.411 | 1.430 | 1.404 | 1.430 | 1.401 |
| bond angles |  |  |  |  |  |  |  |  |
| 8-9-10 | 124.209 | 128.085 | 124.082 | 124.549 | 124.125 | 124.993 | 124.137 | 124.761 |
| 9-10-13 | 99.559 | 106.306 | 100.231 | 100.509 | 100.261 | 101.107 | 100.281 | 101.618 |
| 1-8-9 | 124.120 | 123.801 | 123.928 | 123.902 | 123.952 | 123.910 | 123.956 | 123.767 |
| 7-8-9 | 113.514 | 113.790 | 113.781 | 113.677 | 113.769 | 113.787 | 113.767 | 113.878 |
| 9-10-11 | 110.375 | 102.876 | 110.620 | 110.170 | 110.650 | 109.704 | 110.656 | 109.929 |
| 9-10-12 | 106.419 | 106.446 | 106.535 | 105.876 | 106.543 | 104.878 | 106.545 | 104.237 |
| 10-13-14 | 119.010 | 119.204 | 119.159 | 119.656 | 119.143 | 119.363 | 119.144 | 119.712 |
| 10-13-18 | 119.371 | 119.641 | 119.130 | 119.545 | 119.149 | 119.551 | 119.148 | 119.554 |
| 15-16-19 | 124.699 | 124.435 | 124.835 | 121.811 | 124.830 | 121.983 | 124.832 | 122.441 |
| 17-16-19 | 115.471 | 116.548 | 115.477 | 118.557 | 115.494 | 118.369 | 115.494 | 118.032 |
| 16-19-20 | 114.626 | 114.531 | 114.677 | 130.650 | 114.658 | 130.643 | 114.656 | 130.638 |
| 19-20-21 | 116.210 | 119.573 | 116.719 | 129.901 | 116.774 | 129.647 | 116.784 | 128.305 |
| 20-21-22 | 116.418 | 117.059 | 116.422 | 118.857 | 116.399 | 120.860 | 116.399 | 119.409 |
| 20-21-26 | 125.377 | 124.840 | 125.536 | 122.768 | 125.568 | 122.710 | 125.572 | 122.416 |
| 23-24-27 | 121.608 | 120.807 | 121.693 | 122.569 | 121.682 | 122.419 | 121.684 | 122.181 |
| 25-24-27 | 121.308 | 121.619 | 121.314 | 121.245 | 121.329 | 120.983 | 121.330 | 120.938 |
| 24-27-28 | 121.041 | 121.148 | 121.163 | 121.934 | 121.159 | 121.763 | 121.161 | 121.609 |
| 24-27-29 | 121.272 | 120.859 | 121.429 | 120.788 | 121.450 | 120.753 | 121.454 | 120.525 |
| 31-27-33 | 115.652 | 115.166 | 114.951 | 118.014 | 114.911 | 116.749 | 114.888 | 114.910 |
| 28-27-29 | 117.626 | 117.928 | 117.376 | 118.048 | 117.360 | 118.461 | 117.354 | 118.860 |
| torsion angles |  |  |  |  |  |  |  |  |
| 1-8-9-10 | 35.378 | 30.271 | 34.367 | 30.811 | 33.952 | 30.749 | 33.915 | 30.657 |
| 7-8-9-10 | 147.160 | -151.763 | 148.072 | -151.381 | 148.421 | -151.235 | 148.458 | -151.191 |
| 8-9-10-11 | 76.793 | 75.899 | 70.478 | 69.542 | 70.179 | 69.351 | 70.175 | 69.841 |
| 8-9-10-12 | 54.966 | 45.742 | -59.297 | -57.612 | -59.501 | -57.867 | -59.471 | -59.196 |
| 8-9-10-13 | 169.508 | 169.894 | 174.517 | 176.649 | 174.754 | 176.323 | 174.783 | 175.944 |
| 9-10-13-14 | -106.188 | -90.618 | -104.492 | -107.981 | -104.773 | -108.101 | -104.823 | -106.374 |
| 9-10-13-18 | 74.797 | 87.139 | 76.353 | 73.099 | 76.071 | 73.053 | 76.031 | 74.399 |
| 15-16-19-20 | 1.850 | 0.519 | -2.665 | -1.776 | -2.584 | -1.029 | -2.582 | -0.420 |
| 17-16-19-20 | 178.038 | 179.790 | 177.396 | 178.649 | 177.469 | 178.965 | 177.507 | 179.750 |
| 16-19-20-21 | 179.712 | -178.729 | 179.809 | -175.460 | 179.868 | -172.624 | 179.877 | -170.615 |
| 19-20-21-22 | 179.097 | 174.893 | 179.018 | -174.059 | 179.269 | -175.261 | 179.279 | -176.550 |
| 19-20-21-26 | -0.569 | -5.146 | -0.275 | 1.386 | -0.194 | 3.414 | -0.181 | 3.551 |
| 23-24-27-28 | 1.510 | 7.434 | 1.053 | -2.532 | 0.724 | -1.283 | 0.689 | -1.236 |
| 25-24-27-29 | -1.070 | -3.987 | -0.640 | 0.029 | -0.947 | 1.942 | -0.961 | 0.576 |
| 27-28-30-31 | 176.052 | 176.712 | 176.285 | 175.810 | 176.372 | 176.061 | 176.391 | 176.324 |
| 27-29-32-33 | -176.498 | -175.910 | -176.162 | -176.057 | -176.270 | -176.016 | -176.294 | -175.776 |

**Table S3** Structural parameters in the ground (S_0_) and the charge-transfer excited state (S_2_) of the BS3 compound determined at the time-dependent functional theory PBE0/6-311++G(d,p) level of theory with the inclusion of solvent effects. The bond lengths are given in (Å) and bond and torsion angles in (^o^)

|  | gas phase | | acetone | | DMSO | | water | |
| --- | --- | --- | --- | --- | --- | --- | --- | --- |
|  | S_0_ | S_2_ | S_0_ | S_2_ | S_0_ | S_2_ | S_0_ | S_2_ |
| bond lengths |  |  |  |  |  |  |  |  |
| 3-2 | 1.503 | 1.503 | 1.501 | 1.497 | 1.501 | 1.497 | 1.501 | 1.497 |
| 4-2 | 1.347 | 1.347 | 1.349 | 1.348 | 1.349 | 1.346 | 1.349 | 1.346 |
| 4-5 | 1.333 | 1.334 | 1.335 | 1.332 | 1.335 | 1.332 | 1.335 | 1.332 |
| 6-5 | 1.503 | 1.504 | 1.503 | 1.499 | 1.503 | 1.498 | 1.503 | 1.498 |
| 7-5 | 1.341 | 1.340 | 1.342 | 1.338 | 1.342 | 1.338 | 1.342 | 1.338 |
| 7-8 | 1.332 | 1.334 | 1.334 | 1.337 | 1.334 | 1.335 | 1.334 | 1.335 |
| 8-9 | 1.408 | 1.403 | 1.406 | 1.388 | 1.406 | 1.394 | 1.406 | 1.395 |
| 9-10 | 1.715 | 1.721 | 1.709 | 1.735 | 1.708 | 1.696 | 1.708 | 1.700 |
| 10-11 | 1.461 | 1.462 | 1.466 | 1.470 | 1.466 | 1.467 | 1.467 | 1.467 |
| 10-12 | 1.460 | 1.462 | 1.465 | 1.469 | 1.465 | 1.468 | 1.465 | 1.468 |
| 10-13 | 1.789 | 1.773 | 1.782 | 1.710 | 1.782 | 1.768 | 1.782 | 1.739 |
| 16-19 | 1.416 | 1.375 | 1.416 | 1.403 | 1.415 | 1.402 | 1.412 | 1.404 |
| 19-20 | 1.261 | 1.296 | 1.267 | 1.205 | 1.268 | 1.217 | 1.268 | 1.218 |
| 20-21 | 1.396 | 1.422 | 1.387 | 1.333 | 1.386 | 1.357 | 1.386 | 1.362 |
| 24-27 | 1.375 | 1.387 | 1.364 | 1.356 | 1.363 | 1.348 | 1.363 | 1.349 |
| 27-28 | 1.460 | 1.457 | 1.464 | 1.454 | 1.464 | 1.453 | 1.464 | 1.448 |
| 27-29 | 1.457 | 1.454 | 1.463 | 1.452 | 1.463 | 1.451 | 1.463 | 1.447 |
| 31-32 | 1.448 | 1.435 | 1.452 | 1.437 | 1.453 | 1.436 | 1.453 | 1.438 |
| 32-33 | 1.357 | 1.350 | 1.345 | 1.342 | 1.345 | 1.342 | 1.344 | 1.342 |
| 33-34 | 1.209 | 1.208 | 1.216 | 1.215 | 1.216 | 1.215 | 1.216 | 1.202 |
| 33-35 | 1.499 | 1.497 | 1.499 | 1.492 | 1.499 | 1.492 | 1.499 | 1.491 |
| 35-36 | 1.506 | 1.506 | 1.506 | 1.499 | 1.506 | 1.499 | 1.506 | 1.499 |
| 35-37 | 1.336 | 1.336 | 1.337 | 1.337 | 1.337 | 1.337 | 1.337 | 1.337 |
| bond angles |  |  |  |  |  |  |  |  |
| 8-9-10 | 124.053 | 125.581 | 124.037 | 124.167 | 124.048 | 125.336 | 124.051 | 124.127 |
| 9-10-13 | 99.620 | 100.235 | 100.298 | 103.023 | 99.957 | 101.852 | 100.369 | 101.048 |
| 1-8-9 | 124.063 | 124.313 | 123.939 | 123.475 | 123.935 | 123.537 | 123.936 | 123.827 |
| 7-8-9 | 113.575 | 113.554 | 113.783 | 113.609 | 113.791 | 113.589 | 113.792 | 113.519 |
| 9-10-11 | 110.216 | 109.402 | 110.552 | 108.274 | 110.561 | 106.651 | 110.564 | 106.593 |
| 9-10-12 | 106.435 | 105.886 | 106.459 | 103.399 | 106.469 | 103.385 | 106.472 | 103.345 |
| 10-13-14 | 119.077 | 119.270 | 119.179 | 120.319 | 119.188 | 120.412 | 119.191 | 120.767 |
| 10-13-18 | 119.306 | 119.345 | 119.161 | 119.236 | 119.152 | 119.811 | 119.149 | 119.913 |
| 15-16-19 | 124.704 | 124.358 | 124.859 | 121.672 | 124.87 | 121.689 | 125.089 | 121.774 |
| 17-16-19 | 115.496 | 116.514 | 115.549 | 116.677 | 115.651 | 116.695 | 115.703 | 116.745 |
| 16-19-20 | 114.615 | 115.405 | 114.602 | 125.972 | 114.609 | 125.138 | 114.611 | 124.528 |
| 19-20-21 | 116.250 | 118.211 | 116.864 | 133.932 | 116.895 | 130.533 | 116.904 | 130.394 |
| 20-21-22 | 116.381 | 116.622 | 116.395 | 118.697 | 116.399 | 117.916 | 116.400 | 117.026 |
| 20-21-26 | 125.343 | 124.968 | 125.551 | 121.996 | 125.560 | 122.337 | 125.562 | 122.420 |
| 23-24-27 | 122.096 | 121.181 | 122.281 | 121.643 | 122.289 | 121.337 | 122.292 | 121.475 |
| 25-24-27 | 120.593 | 121.104 | 120.541 | 120.589 | 120.538 | 121.696 | 120.537 | 122.202 |
| 24-27-28 | 121.919 | 122.294 | 122.218 | 121.659 | 122.240 | 121.887 | 122.249 | 121.930 |
| 24-27-29 | 120.324 | 120.122 | 120.544 | 120.318 | 120.563 | 120.046 | 120.570 | 114.685 |
| 28-27-29 | 117.607 | 117.558 | 117.134 | 117.545 | 117.100 | 117.347 | 117.087 | 117.218 |
| 31-32-33 | 116.035 | 115.901 | 117.059 | 115.924 | 117.113 | 115.960 | 117.131 | 115.952 |
| 32-33-34 | 122.623 | 122.381 | 122.866 | 122.830 | 122.880 | 122.802 | 122.885 | 122.749 |
| 34-33-35 | 125.682 | 125.907 | 125.345 | 125.452 | 125.327 | 125.485 | 125.320 | 125.591 |
| 32-33-35 | 116.695 | 111.712 | 111.789 | 111.718 | 111.793 | 111.723 | 111.795 | 111.760 |
| 36-35-37 | 123.645 | 123.655 | 123.494 | 124.034 | 123.489 | 124.060 | 123.488 | 124.076 |
| torsion angles |  |  |  |  |  |  |  |  |
| 1-8-9-10 | 36.411 | 33.502 | 33.335 | 34.301 | 33.267 | 35.632 | 33.242 | 35.352 |
| 7-8-9-10 | -146.214 | -148.993 | -148.977 | -147.385 | -149.037 | -147.409 | -149.040 | -147.921 |
| 8-9-10-11 | 76.560 | 80.676 | 69.112 | 65.160 | 68.838 | 65.504 | 68.760 | 65.917 |
| 8-9-10-12 | 55.219 | 50.177 | 60.527 | 61.536 | 60.697 | 61.320 | 60.742 | 61.240 |
| 8-9-10-13 | -169.726 | -172.194 | -175.841 | -178.245 | -176.043 | -176.185 | -176.099 | -176.109 |
| 9-10-13-14 | -102.042 | -102.642 | -103.142 | -98.902 | -103.021 | -99.991 | -102.987 | -101.869 |
| 9-10-13-18 | 78.791 | 77.501 | 77.612 | 78.038 | 77.725 | 78.186 | 77.756 | 78.261 |
| 15-16-19-20 | 1.321 | 0.476 | -1.223 | 4.941 | -1.301 | 3.613 | -1.340 | 2.314 |
| 17-16-19-20 | -179.135 | -179.659 | 178.661 | 179.015 | 178.607 | 178.689 | 178.579 | 179.831 |
| 16-19-20-21 | -179.821 | -179.482 | 179.977 | -177.194 | 179.986 | -177.91 | 179.993 | -172.036 |
| 19-20-21-22 | -179.729 | 176.677 | 179.865 | -178.284 | 179.915 | -178.252 | 179.933 | -177.229 |
| 19-20-21-26 | 0.614 | -3.342 | 0.164 | -1.836 | 0.201 | -1.895 | 0.214 | -3.149 |
| 24-27-28-30 | -87.293 | -89.890 | -88.877 | -83.681 | -89.079 | -83.095 | -89.163 | -82.453 |
| 27-28-30-31 | 179.964 | 179.144 | -178.510 | 173.888 | -178.415 | 174.075 | -178.374 | 174.580 |
| 28-30-31-32 | -65.202 | -64.898 | -65.453 | -65.123 | -65.511 | -64.911 | -65.534 | 64.832 |
| 30-31-32-33 | 179.603 | 179.953 | 179.740 | 178.181 | 179.734 | 178.234 | 179.720 | 178.324 |
| 31-32-33-34 | -0.910 | -1.164 | -0.373 | -0.026 | -0.342 | -0.003 | -0.334 | -0.027 |
| 31-32-33-35 | 179.032 | 178.730 | 179.592 | 179.914 | 179.616 | 179.945 | 179.622 | 179.894 |
| 32-33-35-36 | -0.800 | -0.546 | -0.662 | -0.533 | -0.640 | -0.660 | -0.627 | -0.612 |
| 32-33-35-37 | 179.307 | 179.656 | 179.319 | 179.485 | 179.336 | 179.300 | 179.349 | 179.176 |
| 34-33-35-36 | 179.140 | 179.343 | 179.302 | 179.406 | 179.317 | 179.286 | 179.327 | 179.607 |
| 34-33-35-37 | -0.753 | -0.455 | -0.718 | 0.576 | -0.706 | -0.754 | -0.696 | -0.805 |

**Table S4** Dipole differences (Δµ_g-CT_) between the excited and the ground state. All values are given in [D]

|  | B3LYP | CAM-B3LYP | LC-BLYP | LC-*ω*PBE | PBE0 |
| --- | --- | --- | --- | --- | --- |
|  | Δ*µ*_g-CT_ | | | | |
| BS1 |  |  |  |  |  |
| gas phase | 6.73 | 6.79 | 7.07 | 7.16 | 6.99 |
| 1.4-dioxane | 7.82 | 7.59 | 8.63 | 8.69 | 8.12 |
| benzene | 7.90 | 8.21 | 8.73 | 8.79 | 8.21 |
| diethylether | 8.14 | 8.55 | 9.17 | 9.21 | 8.46 |
| decanol | 8.36 | 8.86 | 9.58 | 9.61 | 8.69 |
| dichloromethane | 8.38 | 8.89 | 9.64 | 9.66 | 8.72 |
| 1-heptanol | 8.41 | 8.95 | 9.73 | 9.74 | 8.76 |
| 1-hexanol | 8.42 | 8.97 | 9.74 | 9.76 | 8.77 |
| 1-butanol | 8.43 | 9.00 | 9.81 | 9.81 | 8.79 |
| acetone | 8.42 | 8.99 | 9.79 | 9.80 | 8.76 |
| ethanol | 8.43 | 9.01 | 9.82 | 9.83 | 8.78 |
| methanol | 8.42 | 9.00 | 9.81 | 9.83 | 8.77 |
| DMF | 8.50 | 9.11 | 9.95 | 9.95 | 8.86 |
| DMSO | 8.50 | 9.11 | 9.95 | 9.96 | 8.86 |
| water | 8.50 | 9.11 | 9.96 | 9.92 | 8.84 |
| BS2 |  |  |  |  |  |
| gas phase | 7.05 | 6.98 | 7.12 | 7.26 | 7.32 |
| 1.4-dioxane | 8.21 | 8.29 | 8.58 | 8.70 | 8.51 |
| benzene | 8.27 | 8.37 | 8.66 | 8.78 | 8.57 |
| diethylether | 8.59 | 8.72 | 9.08 | 9.19 | 8.90 |
| decanol | 8.86 | 9.05 | 9.46 | 9.57 | 9.19 |
| dichloromethane | 8.90 | 9.09 | 9.51 | 9.61 | 9.23 |
| 1-heptanol | 8.95 | 9.16 | 9.59 | 9.70 | 9.28 |
| 1-hexanol | 8.96 | 9.17 | 9.62 | 9.71 | 9.29 |
| 1-butanol | 9.00 | 9.21 | 9.67 | 9.77 | 9.33 |
| acetone | 8.99 | 9.20 | 9.66 | 9.76 | 9.33 |
| ethanol | 9.01 | 9.23 | 9.70 | 9.79 | 9.35 |
| methanol | 9.01 | 9.22 | 9.69 | 9.79 | 9.34 |
| DMF | 9.09 | 9.33 | 9.79 | 9.90 | 9.41 |
| DMSO | 9.10 | 9.33 | 9.80 | 9.90 | 9.42 |
| water | 9.11 | 9.35 | 9.81 | 9.90 | 9.42 |
| BS3 |  |  |  |  |  |
| gas phase | 6.94 | 6.97 | 7.20 | 7.31 | 7.18 |
| 1.4-dioxane | 7.97 | 8.21 | 8.66 | 8.74 | 8.27 |
| benzene | 8.08 | 8.28 | 8.75 | 8.82 | 8.33 |
| diethylether | 8.24 | 8.58 | 9.15 | 10.09 | 8.56 |
| decanol | 8.45 | 8.89 | 9.54 | 9.59 | 8.79 |
| dichloromethane | 8.48 | 8.93 | 9.60 | 9.65 | 8.82 |
| 1-heptanol | 8.50 | 8.98 | 9.68 | 9.72 | 8.85 |
| 1-hexanol | 8.51 | 8.98 | 9.70 | 9.73 | 8.86 |
| 1-butanol | 8.53 | 9.02 | 9.74 | 9.79 | 8.87 |
| acetone | 8.50 | 9.00 | 9.74 | 9.78 | 8.85 |
| ethanol | 8.51 | 9.02 | 9.76 | 9.80 | 8.86 |
| methanol | 8.50 | 9.00 | 9.76 | 9.80 | 8.93 |
| DMF | 8.55 | 9.12 | 9.88 | 9.92 | 8.94 |
| DMSO | 8.55 | 9.12 | 9.89 | 9.93 | 9.00 |
| water | 8.56 | 9.13 | 9.91 | 9.94 | 9.01 |
